# ClinSegNet: Towards Reliable and Enhanced Histopathology Screening

**DOI:** 10.3390/bioengineering12111156

**Published:** 2025-10-25

**Authors:** Boyang Yu, Hannah Markham, Karwan Moutasim, Vipul Foria, Haiming Liu

**Affiliations:** 1 School of Electronics and Computer Science (ECS), University of Southampton, Southampton SO17 1BJ, UK; by3n24@soton.ac.uk; 2 Cellular Pathology Department, University Hospital Southampton NHS Foundation Trust, Southampton SO16 6YD, UK; hannah.markham@uhs.nhs.uk (H.M.); karwan.moutasim@uhs.nhs.uk (K.M.); vipul.foria@uhs.nhs.uk (V.F.); 3 Southampton Research and Clinical Histopathology Samples Team (SRCH Team), University Hospital Southampton NHS Foundation Trust, Southampton SO16 6YD, UK

**Keywords:** histopathology segmentation, clinical screening, deep learning, high recall, human-centric framework

## Abstract

In histopathological image segmentation, existing methods often show low sensitivity to small lesions and indistinct boundaries, leading to missed detections. Since, in clinical diagnosis, the consequences of missed detection are more serious than false alarms, this study proposes ClinSegNet, a recall-oriented and human-centred framework for reliable histopathology screening. ClinSegNet employs a composite optimisation strategy, termed HistoLoss, which balances stability and boundary refinement while prioritising recall. An uncertainty-driven refinement mechanism is further introduced to target high-uncertainty cases with limited fine-tuning cost. In addition, a clinical data processing pipeline was developed, where pixel-level annotations were automatically derived from IHC-to-H&E mapping and combined with public datasets, enabling effective training under limited clinical data conditions. Experiments on the NuInsSeg and NuInsSeg-UHS datasets showed that ClinSegNet achieved recall scores of 0.8803 and 0.8917, further improved to 0.8983 and 0.9053 with HITL refinement, while maintaining competitive Dice and IoU. Comparative and ablation studies confirmed the complementary design of the framework and its advantage in capturing small or complex lesions. In conclusion, ClinSegNet provides a clinically oriented, recall-prioritised framework that enhances lesion coverage, reduces the risk of missed diagnosis, and offers both a methodological basis for future human-in-the-loop systems and a feasible pipeline for leveraging limited clinical data.

## 1. Introduction

In recent years, with the rapid development of artificial intelligence in medical imaging, automatic analysis of histopathological images has become a key research direction. Traditional pathological diagnosis relies heavily on the manual judgment of pathologists, which is time-consuming, labour-intensive, and subjective, and can be affected by empirical differences. Deep learning, especially Convolutional Neural Networks (CNNs), has demonstrated promising performance in image segmentation tasks, offering new possibilities for automated pathological image analysis.

Among many segmentation models, U-Net and its derivatives are widely used in various semantic segmentation tasks of pathological images due to their excellent performance in medical image scenarios. However, in real clinical applications, tissue sections are often derived from different organs, and their morphological, staining, and structural characteristics vary significantly. Meanwhile, lesion regions usually occupy a small proportion of the image, with fuzzy and irregular boundaries, which brings significant challenges to accurate localisation.

To address these challenges, academia and industry continue to explore more robust segmentation frameworks to support high-risk application scenarios such as clinical screening and auxiliary diagnosis. In this context, this study proposes ClinSegNet, a clinically oriented and recall-prioritised segmentation framework designed for multi-organ histopathology images. The aim of ClinSegNet is to improve adaptation across tissues, enhance recall of lesion regions, and provide a methodological foundation for future human-centred diagnostic systems.

Although existing deep segmentation models have achieved high accuracy on public medical datasets, three key limitations remain:

- **Lack of tissue awareness:** Most methods do not consider the tissue source of the input images. The model cannot distinguish structural patterns from different organs during training. This “tissue insensitivity” leads to unstable performance under multi-organ mixed training and limits generalisability.
- **Low recall of lesion regions:** Most objectives are dominated by pixel accuracy or the Dice coefficient, neglecting recall. In clinical screening, missing a potential lesion is more harmful than false detection. Improving recall is, therefore, critical, even at the expense of some precision.

Accordingly, ClinSegNet was designed with three objectives: organ-aware perception, boundary sensitivity, and expert-guided refinement. These objectives are outlined as follows:

1. **Organ embedding mechanism:** Organ label information was integrated into the model, enabling conditional modulation of different tissues during feature extraction and improving cross-organ generalisation.
2. **Edge-assisted branch:** Edge supervision was introduced to strengthen boundary localisation, reducing lesion blurring and breakage, and improving contour consistency.
3. **Recall-prioritised loss function (HistoLoss):** A composite loss combining Tversky and binary cross entropy (BCE) was formulated to bias optimisation towards recall while maintaining training stability.
4. **Expert-guided refinement:** An uncertainty-driven Human-in-the-Loop (HITL) strategy was adopted to selectively fine-tune high-uncertainty cases, simulating expert correction in clinical workflows.

The main contributions of this study are summarised as follows:

- Proposed **ClinSegNet**, a clinically oriented, recall-first segmentation framework for histopathology images.
- Designed **HistoLoss**, a composite loss function combining BCE and Tversky, to ensure stable optimisation while prioritising recall.
- Incorporated organ-aware embedding and edge-assisted supervision into a SEU-Net backbone, enhancing cross-organ discrimination and boundary localisation.
- Introduced a human-centred refinement strategy based on uncertainty-driven HITL, demonstrating that even limited recall improvements hold clinical value.
- Established a practical **clinical data processing pipeline**, where pixel-level annotations were automatically derived from IHC-to-H&E mapping and then integrated with public datasets, enabling effective training under limited clinical data conditions.

## 2. Literature Review

### 2.1. Medical Image Segmentation

Medical image segmentation aims to accurately divide different anatomical structures, tissues, or lesion regions in medical images, which is the basic link of core tasks such as Computer Aided Diagnosis (CAD), treatment planning, and disease progression monitoring [1,2]. Through segmentation, clinicians are able to obtain quantitative morphological information, such as the size, shape, and location of the lesion, as well as its relationship with surrounding tissues, which plays an irreplaceable role in disease screening, preoperative planning, radiotherapy dose calculation, and long-term follow-up [3,4]. In the past decade, with the rapid development of deep learning, automated segmentation techniques have made significant progress in various image modalities, but the model performance largely depends on the matching degree between data features and task scenes [5].

Medical imaging can be divided into two main categories:

- **Radiological Imaging**, such as computed tomography (CT), magnetic resonance imaging (MRI), and Ultrasound, is commonly used to visualize anatomical structures and detect lesions at a macroscopic scale. Such images have relatively stable imaging parameters and low resolution variation, but may be affected by noise, motion artefacts, and signal loss caused by metal implants [6].
- **Microscopy-based Imaging**, including Histopathology and Cytology imaging, can reveal cellular structure and tissue morphological characteristics at the microscopic level. Such images have extremely high resolution and rich details, accompanied by significant colour differences, complex textures, and morphologically diverse nuclear and tissue structures. Different modalities put forward different requirements for the input scale, feature extraction strategy, context information modelling, and generalization ability of the segmentation model [7].

Among them, histopathological image is regarded as one of the most challenging modalities in the segmentation task due to its fine-grained characteristics and high-dimensional data scale, which lays a foundation for the research background focused in the subsequent sections.

In the early studies on medical imaging segmentation, conventional techniques primarily incorporate rule-based or manual feature designing approaches like thresholding [8,9], region growing [10], active contours [11], watershed algorithms [12], and Graph Cuts [13]. These approaches consist of the utilisation of low-level information to establish the segmentation patterns, which is the core principle behind these methods. In most cases, the low-level features used for the purpose are image edge gradient and texture features. This way, the proposed strategies work only in some specific tasks that involve good imaging conditions established in advance and well-defined target boundaries. Furthermore, they are very sensitive to noise or changes in lighting, as well as anatomical structures with different morphologies, and can have lower adaptability across different imaging modalities [2]. Those techniques require tedious efforts of feature redesigning and parameter tuning to guarantee high performance. Accordingly, it is difficult to achieve the task of maintaining the stability of performance and generalization under complex clinical circumstances. Secondly, they usually lack end-to-end learning capability and exhibit difficulty in capturing the rich diverse patterns hiding in the data.

With the development of deep learning, especially the success of Convolutional Neural Network (CNN) in medical image analysis, end-to-end segmentation networks have quickly become mainstream. Among them, the U-Net architecture is widely regarded as a landmark model for medical image segmentation [14]. Its symmetric encoder–decoder structure combined with skip connections effectively integrates shallow spatial details and deep semantic information, and significantly improves segmentation performance in multiple medical image modalities [14]. Based on the success of U-Net, a large number of derivative architectures have emerged: for example, UNet++ introduced dense connections to alleviate the semantic gap [15], Attention U-Net enhanced feature selectivity through the channel and spatial attention mechanism [16], and TransUNet added a Transformer encoder before the decoder to realize global dependency modelling [17]. In the direction of automation and generalisation, nnU-Net achieved stable optimal performance on multiple datasets by adaptively configuring the network structure and hyper-parameters [18]. For efficiency and deployment requirements, lightweight variants such as UNeXt combined convolution and MLP-Mixer modules to significantly reduce the number of parameters while maintaining high accuracy [19]. In addition, MedT combined with vision Transformer structure shows unique advantages in global context modelling and cross-domain generalisation [20]. These methods exhibited higher robustness when they dealt with multi-scale structures, complex backgrounds, and weak boundaries. And they also provided a solid foundation for model optimisation for specific tasks, such as histopathology segmentation.

Following these developments, Transformer-based segmentation architectures such as Swin-UNet [21], UNETR [22], and TransFuse [23] further extended the U-Net paradigm, leveraging hierarchical self-attention to capture long-range dependencies while preserving local feature precision. These approaches demonstrated improved robustness in multi-scale medical and histopathological segmentation, marking a key shift from convolution-dominant designs to hybrid or Transformer-driven frameworks.

In general, deep learning methods significantly improved the automation and robustness of medical image segmentation, and achieved excellent performance in multiple image modalities. However, histopathological images are still considered to be the most challenging modality in segmentation tasks due to their fine-grained features and high-dimensional data scale, which will be the focus of subsequent research.

### 2.2. Histopathology Image Segmentation Based on Deep Learning

The segmentation of histopathology images presents unique challenges arising from complex tissue structures and diverse imaging conditions. Being different from radiological images, pathological images often present highly variable colour distributions, irregular cell arrangements and blurred boundaries. These factors can also be affected by artifacts during tissue preparation and scanning. Therefore, histopathological segmentation requires high accuracy and strong robustness under staining protocols, organ types, and disease categories.

The early representative work HoVer-Net proposed by Graham et al. introduced the Horizontal and vertical distance (HoVer) branch, which used the horizontal and vertical distance mapping from pixels to the centroid of the nucleus to separate the overlapped nucleus, and added the type prediction branch to realize simultaneous instance segmentation and classification [24]. It achieved the best segmentation performance on multi-tissue datasets like PanNuke [25], which reported Dice 0.785 [24]. Subsequently, the Generalised Framework was applied to the multi-cancer WSI segmentation [26]. The Generalised Framework adopted an integrated strategy of DenseNet-121 [27], Inception-ResNet-V2 [28] and DeepLabV3+ [29] and combined with efficient patch sampling and class-balanced training. Furthermore, Kim et al. addressed the memory bottleneck issue in the inference of high-resolution WSI by proposing a method that introduces principal component analysis (PCA) and discrete wavelet transform (DWT) in the compressed domain, and combines the reconstruction results with Wavelet Weighted Ensemble [30]. This framework achieved an average Dice score 0.804 in the segmentation of colorectal cancer WSI, which represented a 2.7% improvement compared to the spatial domain method [30].

In the improvement line of HoVer-Net, TSHVNet incorporated Transformer attention and the SimAM channel-spatial attention module into both the backbone and branch networks, aiming to strengthen global dependency modelling and emphasis core regions [31]. Moreover, MulverNet further enhanced HoVer-Net by integrating Multiple Filter Units and Attention Gates so that improve multi-scale feature integration and extraction while reducing irrelevant noise [32]. In a parallel direction, MIU-Net embedded an improved Inception module and a Mix-Attention mechanism into the U-Net backbone to boost fine-grained feature extraction for complex boundaries [33].

Recently, models have further evolved towards efficient inference and deployment friendliness. HoverFast compressed HoVer-Net into a lightweight student model through knowledge distillation and optimized parallel processing, data reading and writing, and post-processing on an engineering implementation, reducing 40× WSI inference time from 2 h to 6 min and maintaining an average Dice = 0.91 consistent with the original HoVer-Net [34]. What’s more, FRE-Net proposed a Full-Region Enhanced strategy, which used the global context information of the nucleus and background at the same time, and introduced a contour detection branch to strengthen the boundary representation [35]. Furthermore, HistoNeXt combined ConvNeXt encoder with dual-mechanism feature pyramid fusion to integrate three branches, kernel pixel (NP), horizontal and vertical distance (HV), and type prediction (TP) into the same decoder system [36]. It achieved Dice = 0.874 CPM17 [7] dataset, and the performance is better than Transformer-like methods [36].

Moreover, recent studies have introduced several Transformer-based architectures tailored for histopathological image segmentation, aiming to capture long-range dependencies beyond the capability of CNNs. SwinCup [37] embeds the Swin Transformer into a cascaded upsampling framework for gland segmentation, enabling hierarchical attention across tissue scales and achieving superior boundary accuracy on the GLAS and CRAG datasets. MTPA-Net [38] employs a multi-scale Transformer with parallel attention modules to enhance contextual learning for nuclei segmentation, effectively preserving structural consistency in densely packed cellular regions. Imran et al. proposed a Transformer-based framework for multi-class skin cancer segmentation, integrating hierarchical attention and uncertainty visualization to improve interpretability and diagnostic reliability [39]. Together, these works highlight the growing role of Transformer architectures in pathology, demonstrating their strength in modelling global context, enhancing boundary sensitivity, and improving clinical interpretability, but they still face challenges such as scarce data, high computational costs, and difficulties in boundary identification in segmentation tasks [40].

These studies show that the technical route of histopathology segmentation is gradually evolving from the integration of segmentation and classification tasks to multi-scale feature fusion and global context modelling plus inference efficiency optimization, and the performance improvement is continuously verified on public benchmarks by Dice or DSC and other indicators. Although recent histopathology image segmentation techniques continuously introduce complex mechanisms such as multi-branch, multi-scale fusion and global context modeling in architecture design, thereby achieving high Dice or DSC scores on public benchmarks, this trend also exposes obvious shortcomings. Most studies focus more on improving the comprehensive accuracy in the process of model optimization, and pay insufficient attention to the high recall rate, which is crucial in clinical screening tasks [41,42]. In practical applications, the risk of missing positive cases is often more serious than false detection, and current methods may sacrifice sensitivity while pursuing overall accuracy. Thus it weakens their utility in early detection of high-risk lesions. This gap highlights the need to introduce a high-recall optimization objective into model design and evaluation. In addition, multi-organ segmentation also further challenges due to domain shift across tissues and staining methods, where existing methods show limited generalization [43,44].

### 2.3. Human-in-the-Loop (HITL) Approaches in Medical AI

With the wide application of deep learning in the field of medical images, how to achieve efficient, safe, and interpretable models under a human–computer collaboration framework has gradually become a research hotspot. As a method of incorporating expert knowledge into the model training and inference process, Human-in-the-Loop (HITL) has shown great value in medical image analysis and auxiliary diagnosis tasks in recent years.

Budd et al. systematically reviewed the application of HITL in medical image analysis from the perspective of Active Learning, emphasizing its central role in selecting the most informative samples, introducing interactive feedback mechanisms, and reducing data labelling overhead [41]. At the same time, it points out that the strategy has unique advantages in ensuring medical security and they further proposed that HITL is expected to alleviate the problems of data scarcity and training instability, and it is a key research direction to achieve data efficiency improvement and robustness enhancement in medical image analysis [41]. Wang et al. further extended this direction by exploring the integration of deep active learning with semi-supervised and transfer learning strategies, demonstrating the potential of the HITL framework to optimise performance and save manual annotation costs in medical image tasks [45]. Moreover, Holzinger et al. emphasised the role of HITL in explainable artificial intelligence (XAI), especially in the scenario of digital pathology, where it not only participates in diagnostic judgment, but also intervenes in the interpretation process, helping to improve the causal reasoning ability and clinical confidence of the model [46].

In terms of specific applications, Gu et al. designed the Impetus system, which deeply integrates AI reasoning and feedback from human pathologists into the digital pathology process, and which demonstrated the user experience and interaction design elements that need to be concerned with when the HITL framework is deployed [47]. Zhang et al. focused on the task of sepsis diagnosis in intensive care and built the SepsisLab system, which uses uncertainty visualization, diagnostic hypothesis generation, and expert interactive test recommendations to significantly improve the confidence and clinical adaptability of AI systems in high-risk medical scenarios [48].

In addition, Slany et al. proposed the CAIPI-in-Practice framework to introduce an interactive interface to a medical image classification task, enabling experts to correct the model’s predictions and interpretations, achieving almost 97% accuracy, even when only using a small number of interactive examples, while reducing the annotation effort by 80% [49]. Furthermore, it was found that HITL is especially suitable for cases where the model is error-prone in weak staining areas—such as Ki-67 index analysis in breast cancer tissue—and expert feedback can effectively correct the critical errors, thereby improving the classification accuracy and the credibility of pathological diagnosis [50].

Although the above studies comprehensively demonstrate the potential of HITL framework to improve the safety, interpretability, and efficiency of medical AI systems, there are still some important research limitations. First, most existing works mainly focus on classification tasks or decision support in structured scenes, and pay less attention to pixel-level spatial reasoning tasks such as histopathological image segmentation. However, in the actual pathological diagnosis, the characteristics of image boundaries are fuzzy and the lesion area is small, which puts forward higher accurate positioning and fine-grained learning requirements for the model. Secondly, although interactive correction or active query mechanisms have been introduced in previous studies, many existing systems still rely on fixed labelling protocols or post-hoc feedback mechanisms, and have not yet realized real dynamic collaboration and real-time human–machine co-construction model process. For example, although the CAIPI framework allows for experts to correct model interpretation, its interaction mode is still relatively limited, and it lacks modelling support for contents such as “tissue-specific preference”. At the same time, the existing HITL schemes generally aim at the overall performance optimisation and rarely focus on the more critical optimisation of “high recall” in the clinical process, which is exactly the core index that affects the diagnostic safety in the actual lesion screening.

### 2.4. Research Gap and Motivation

In summary, the existing research forms a relatively complete technical system in the field of medical image segmentation, which has gradually developed from traditional methods to end-to-end architectures based on deep learning, and shows excellent performance across a variety of image modalities. On this basis, research on pathological image segmentation has also evolved from case segmentation to multi-scale fusion, global context modelling, and inference efficiency optimisation. However, there are still some important research gaps:

1. **Ignoring recall:** Most existing studies use Dice or IoU as the main evaluation metric, while Recall is often weakened or even not explicitly reported, although it is more critical in clinical safety.
2. **Risk of missed detection:** Due to excessive focus on overall accuracy, current methods tend to miss lesions that are small in size or difficult to detect, thus undermining their utility in early screening tasks.
3. **Limited HITL focus:** Current Human-in-the-Loop (HITL) methods are mainly applied to classification tasks, annotation optimization, or diagnostic assistance, and pay little attention to pixel-level segmentation tasks.
4. **Lack of recall-oriented HITL strategies:** Existing HITL frameworks explicitly introduce Recall optimization, which is precisely the key to ensure the reliability of diagnostic segmentation of pathological images.

Therefore, the current research status reveals a significant gap: how to effectively introduce human-machine collaboration mechanisms into histopathology segmentation, and take high recall as the optimisation core, so as to balance clinical safety and model practicality. To address this problem, this paper proposes a high-recall pathological image segmentation method guided by organ conditions, and integrates edge-aided supervision with a human–machine collaborative optimisation mechanism to explore a feasible path to improve sensitivity and interpretability in complex clinical scenarios.

Building upon the above motivation, this work further focuses on addressing the architectural aspect of recall-oriented segmentation. Although existing techniques such as Squeeze-and-Excitation (SE) blocks, Feature-wise Linear Modulation (FiLM), and edge-assisted supervision have been individually explored in prior works, they have rarely been integrated under a unified framework for recall-oriented histopathology segmentation. The proposed ClinSegNet uniquely combines these three mechanisms in a task-driven manner: FiLM embeddings introduce organ-aware contextual conditioning to adaptively modulate features across tissue types; SE blocks enhance channel-level discrimination within this conditioned space; and the edge branch constrains boundary prediction to mitigate false negatives in metastatic regions. This synergistic design enables ClinSegNet to achieve both high recall and fine boundary delineation, offering a clear advantage over conventional architectures such as U-Net [14], Attention U-Net [16], and HoverNet [24].

## 3. Methods

### 3.1. Datasets and Pre-Processing

#### 3.1.1. NuInsSeg Dataset

The NuInsSeg dataset is an open source dataset for tissue histopathology image segmentation, including various organs and tissue types. Its original data includes both human and mouse tissue sections and provides pixel-level semantic segmentation annotations. Considering the aim of this project, this study only uses the human tissue part, covering more than twenty organs tissues. As summarised in Table 1, the dataset covers a variety of human organs and tissues.

**Figure 1:**
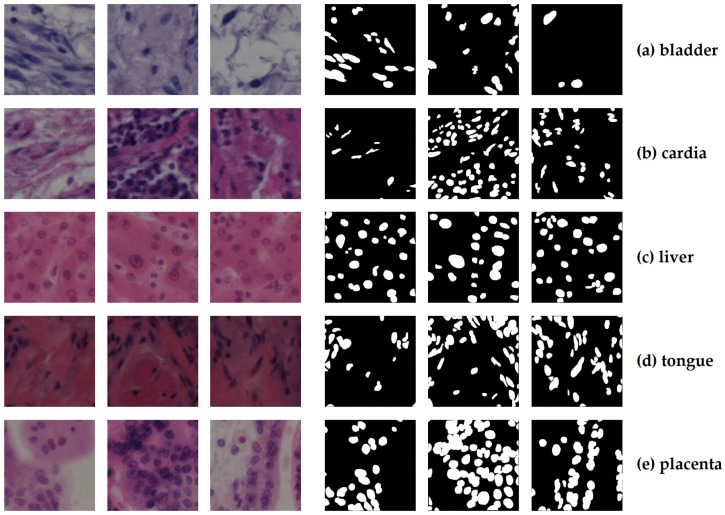
Representative examples from the NuInsSeg dataset, illustrating variations in tissue morphology and staining across different organs.

In detail, this dataset contains approximately 472 high-resolution tissue section images (with a patch size of 512 × 512 pixels) and provides semantic segmentation masks for each image. The annotation is finished manually to ensure the accuracy of the tissue boundaries. As shown in Figure 1, the data shows that there are significant differences in morphological features and staining performances between different organs and tissues. Even if the number of images is basically balanced, these cross-organ differences still significantly increase the complexity of the segmentation task.

There are four reasons for selecting the NuInsSeg dataset as a benchmark. Firstly, it covers multiple organ tissue slices, which enables a systematic evaluation of cross-organ generalisation. Secondly, as an open-source dataset, it ensures the reproducibility of experimental results. Thirdly, the dataset presents significant challenges in terms of staining differences, structural heterogeneity, and class imbalance, which are critical for validating the robustness of segmentation models. Finally, NuInsSeg remains one of the few histopathology benchmarks that are accessible, since many previously released datasets are no longer publicly available due to the sensitive nature of clinical pathology images.

##### Pre-Processing

In order to increase data availability and training stability, pre-processing operation was implemented to the NuInsSeg dataset. Starting by applying standard data augmentation techniques to every image, such as colour perturbation and flipping both horizontally and vertically. When datasets are small, these adjustments can increase the number of training samples and reduce the risk of overfitting. The second step is that all images are converted to greyscale in order to eliminate the potential impact of variations in staining conditions of different tissues so that model can focus on structural characteristics rather than staining variance.

Images were initially arranged by organ/tissue category for dataset partitioning in order to guarantee that each class had representative samples in both training and testing. The dataset was split into training and testing sets in a ratio of 8:2. The training set was used for model training and optimisation, and the testing set was used for performance evaluation. For the reproducibility, a fixed random seed was applied during dataset splitting and kept consistent throughout all experiments. Table 2 summarises the comparison of data volume before and after pre-processing.

**Figure 2:**
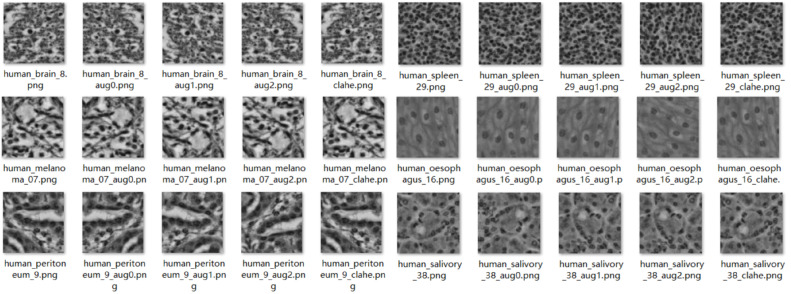
Representative samples from the NuInsSeg dataset after pre-processing, showing examples of different organs/tissues and augmented images.

Moreover, for organ/tissue name embedding and fast indexing during training, image files of different categories adopt a uniform naming convention as shown in Figure 2. All files are named in the “human_organ _id” format. For example, the image from the kidney is named “human_kidney_id”, and the image from the lung tissue is named “human_lung_id”. The naming scheme guarantees that the encoded filenames provide word embeddings which enable the model to differentiate between organs and tissues.

#### 3.1.2. UHS-MelHist Dataset

**UHS Melanoma Histopathology (UHS-MelHist) Dataset** is derived from the pathological sections of melanoma lymph node metastases in real clinical practice, and covers two staining modalities: hematoxylin-eosin staining (H&E) and Melan-A immunohistochemical staining (Melan-A-IHC). Each sample contains a pair of stained images, which show the tissue structure and immune marker expression, respectively, to assist in identifying the metastatic area.

The original images were manually cropped from Whole Slide Images (WSI) by the hospital pathology department to obtain the Region of Interest (ROI). Unlike raw Whole Slide Images (WSI), each sample in the UHS-MelHist dataset was obtained from pre-screened tissue regions manually selected by pathologists from metastatic lymph node slides. Unlike raw Whole Slide Images (WSI), each sample in the UHS-MelHist dataset was obtained from pre-screened tissue regions manually selected by pathologists from sentinel lymph node (SLN) sections that had been histologically confirmed as melanoma metastases using Melan-A immunohistochemistry. The pathology department performed an initial cropping on the WSI to isolate diagnostically relevant fields before providing them for research use. Based on these pre-selected areas, we further extracted 37 paired ROIs from 6 representative SLN slides, each of approximately 2100×1500 pixels under 10× magnification as shown in Table 3. At this magnification, both Melan-A positive areas and surrounding normal tissue structures can be clearly distinguished, enabling reliable identification of metastatic regions without requiring higher-resolution scanning. This design ensures that the dataset retains sufficient morphological detail for segmentation tasks while maintaining computational efficiency.

**Figure 3:**
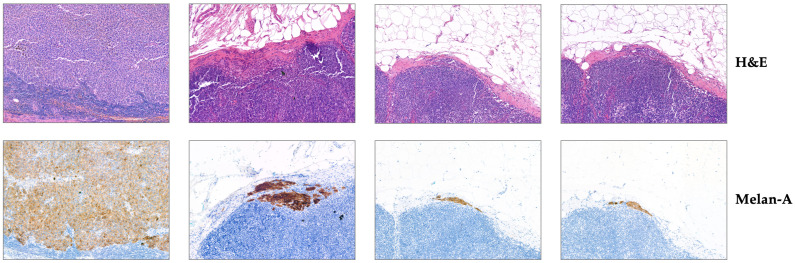
Representative examples from the UHS-MelHist dataset. Each ROI was manually cropped from Whole Slide Images (WSI).

**Figure 4:**
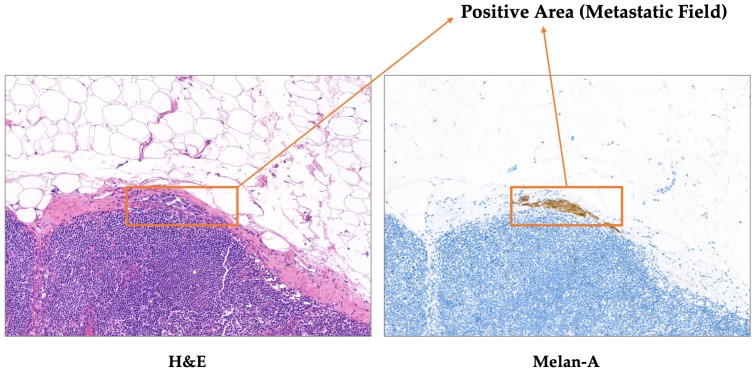
Example highlighting metastatic region in UHS-MelHist dataset. The H&E image (**left**) shows tissue morphology where the metastatic region (orange box) is difficult to distinguish from the surrounding structures. The corresponding Melan-A image (**right**) highlights melanocytic cells with positive brown staining in the same area, which made the metastatic field clearly visible.

All images are provided in pairs to ensure a one-to-one correspondence between H&E and Melan-A-IHC stained images to facilitate subsequent registration and comparative analysis. H&E staining highlights the general tissue morphology, while Melan-A immunohistochemistry specifically labels melanocytic cells, facilitating the identification of metastatic regions. Representative paired images from the UHS-MelHist dataset are shown in Figure 3, illustrating both H&E and Melan-A staining for the same regions of interest. And Figure 4 highlights an example where the metastatic field is indistinct on H&E, but clearly visible on Melan-A staining.

The tumour regions contained in this dataset show significant structural heterogeneity in morphology, including metastases of varying sizes, irregular border contours, and lesion regions with uneven staining. In some samples, the tumour cells and surrounding normal tissues are highly mixed, and the boundaries are fuzzy or even invisible, which brings great challenges to the segmentation task. In addition, some tissue regions have significant inflammatory infiltration, necrosis, or lymphoid tissue overlap, further increasing the difficulty of model discrimination. Overall, the data is highly close to the real clinical environment, and can effectively simulate the actual difficult situation in pathological image segmentation.

The data was provided by the **University Hospital Southampton (UHS)**, fully anonymised prior to transmission, and securely transmitted to the project leader via the Soton SafeSend platform.

##### Pre-Processing

**Figure 5:**
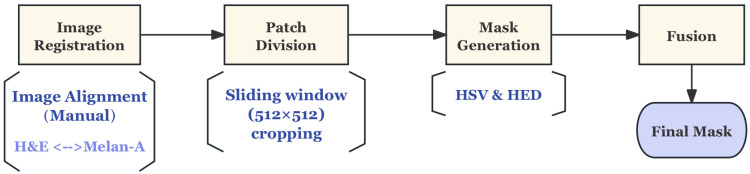
Pre-processing pipeline for the UHS-MelHist dataset.

The UHS-MelHist dataset has a relatively complex pre-processing process compared with NuIns-Seg, mainly because it does not provide ready-made segmentation labels, which were generated automatically using paired Melan-A stained images. The whole process includes registration, patch division, mask generation, and fusion as shown in Figure 5.

**Figure 6:**
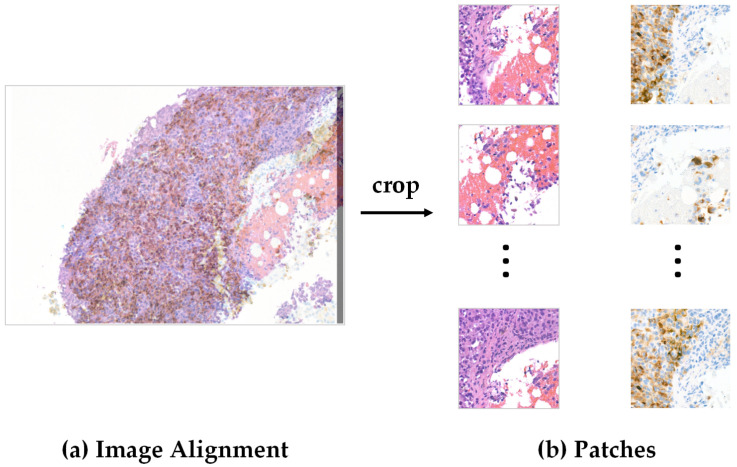
Example of UHS-MelHist pre-processing, showing (**a**) image alignment of paired H&E and Melan-A slides. (**b**) The cropped patch pairs.

Firstly, the paired H&E and Melan-A images are not perfectly aligned at the original ROI level, so the Melan-A images need to be rotated and translated by manually set parameters during pre-processing to ensure the spatial correspondence with the H&E images. Subsequently, 512 × 512 patches with a step size of 256 pixels were cut in a sliding window fashion within the overlapping region to obtain paired H&E/Melan-A patches, as shown in Figure 6.

**Figure 7:**
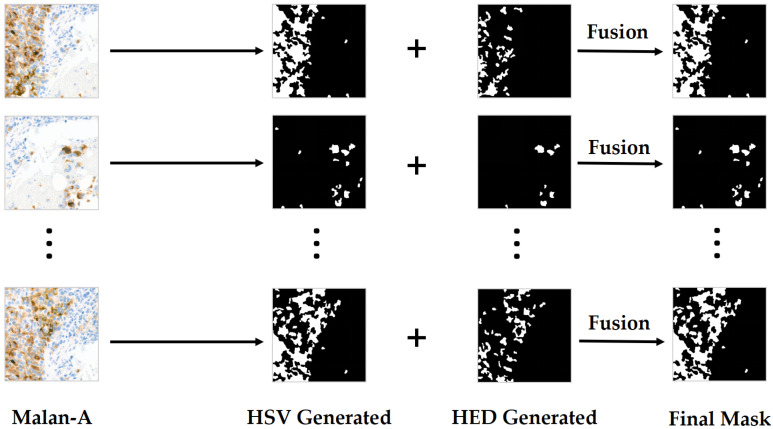
Mask generation pipeline for the UHS-MelHist dataset. Candidate masks are derived from Melan-A images using HSV-based and HED-based methods, and then fused to obtain the final mask.

To generate usable segmentation labels from Melan-A images, two complementary mask extraction methods are designed in this study. The first one is based on HSV colour space, uses the hue range to separate the typical DAB yellow-brown signal, and excludes the background blue area. After morphological operation and small area filtering, the preliminary mask is obtained. The second one is based on HED pigment decomposition, which separates DAB channels from Melan-A images, and combines the Gaussian smoothing and Otsu thresholding methods to obtain a refined mask, which is then optimized by opening and closing operations and small-region filling. Finally, the two candidate masks were fused by logical OR (union) to obtain a more robust label. The mask generation procedure is illustrated in Figure 7.

This series of operations not only ensures the spatial alignment of H&E and Melan-A images at the patch level, but also provides high-quality labels for H&E images. Therefore, the pre-processing process of UHS-MelHist is more complex than that of public datasets, but it enables the model to effectively learn the segmentation features of metastatic lesion regions without manual annotation.

In order to improve the robustness of the model and reduce the risk of overfitting on the relatively small-sample-size UHS-MelHist dataset, standard data augmentation operations are performed on the trimmed paired patches. All enhancements were aligned on H&E to mask to ensure spatial correspondence.

Enhancement strategies include random rotations (±90°), horizontal and vertical flips, and mild colour perturbations (fine tuning of brightness, contrast, and saturation) applied to the H&E image, and the images were converted to greyscale image, consistent with data augmentation of NuInsSeg dataset. With the above enhancement, the amount of data is shown in Table 4, which greatly improved the diversity of training data.

#### 3.1.3. Data Usage Strategy

**Figure 8:**
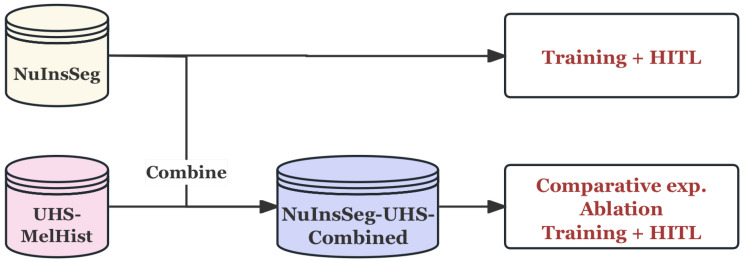
Dataset usage strategy in this study.

In this study, these two datasets played different roles in experiments: NuInsSeg was used for independent training with benchmark validation, while UHS-MelHist was merged with NuInsSeg for larger scale experiments. The data workflow is shown in Figure 8.

Firstly, as a public multi-organ benchmark dataset, NuInsSeg is used for the initial training of the model and the preliminary verification of the Human-in-the-Loop (HITL) process. Its diversity across organs helps test the performance on generalization ability and provides benchmark results for subsequent experiments.

Then, considering the amount of data of UHS-MelHist dataset, there was no separate experiments implemented on this, but it combined with the NuInsSeg dataset after unified pre-processing and naming convention to construct a comprehensive dataset **NuInsSeg-UHS-Combined**. The training and HITL process, comparison experiment, and ablation experiment were carried out on this dataset.

The construction of this comprehensive dataset not only expanded the data scale, but also enhanced the clinical relevance of the experiment, avoiding the bias caused by the limited size of a single dataset.

### 3.2. Network Architecture

**Figure 9:**
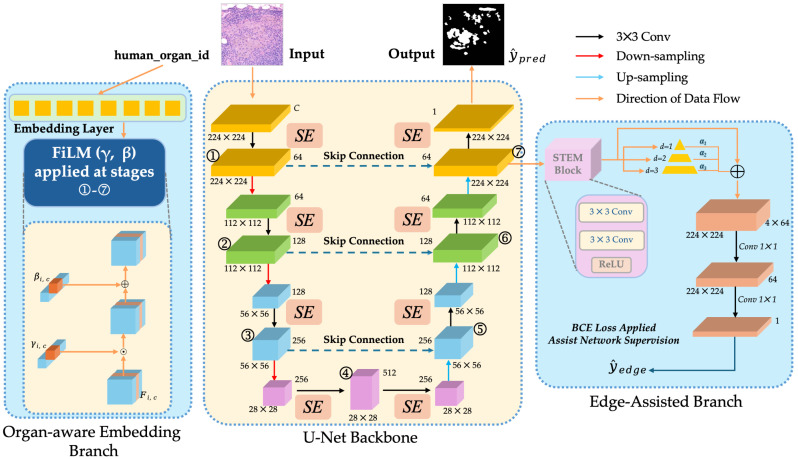
Overall architecture of ClinSegNet, consisting of an SEU-Net backbone, an organ-aware FiLM embedding branch, and an edge-assisted supervision branch.

The backbone network in this study is based on the classical U-Net structure and has been modified for the characteristics of histopathological image segmentation. The overall design goal is to achieve high recall in the lesion area, that is, to ensure the segmentation performance while minimising missed detection, so as to meet the requirements of sensitivity in clinical applications. To this end, the model introduces the SE module, organ-conditional embedding, and edge-assisted branching on the basis of U-Net to form an organ-aware and boundary-enhanced segmentation network, and the overall structure is shown in Figure 9.

#### 3.2.1. SEU-Net Backbone

ClinSegNet adopts a U-Net–based [14] backbone enhanced with SE blocks (SEU-Net) to recalibrate channel responses, and its core consists of a symmetric encoder–decoder. The encoder gradually extracts high-level semantic features through convolution and downsampling, and the decoder gradually restores the spatial resolution through upsampling and fuses the shallow detail features with the deep semantic features through skip connections. This structure has been widely used in medical image segmentation tasks due to its ability to capture the global context and preserve local boundary information.

**Figure 10:**
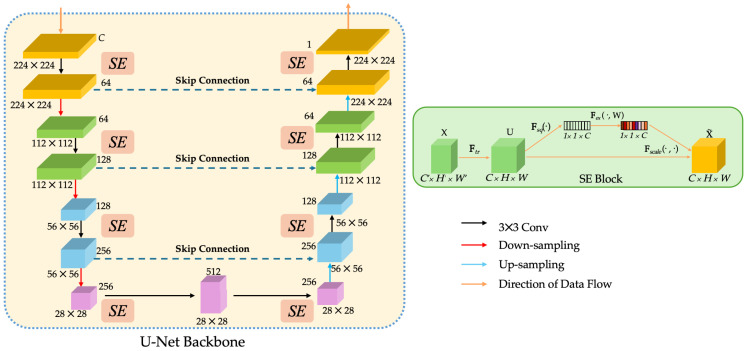
The U-Net backbone integrated with SE blocks (SEU-Net). SE blocks are inserted into each encoder and decoder stage to recalibrate channel responses.

However, in cross-organ pathological images, there are significant differences in tissue structure and staining performance, resulting in complex feature distribution and containing a significant amount of redundant information. Relying solely on the convolution operation of U-Net, it is difficult to effectively highlight the important channels of the lesion area in multi-scale features. Motivated by this challenge, this study introduces Squeeze-and-Excitation (SE) module [51] into the encoder and decoder stages of U-Net. The SE block models the inter-channel dependencies by global average pooling and uses the fully connected layer to generate the weight vector to realise the channel-by-channel recalibrations of the feature map. As shown in Figure 10, SE blocks are embedded into each stage of the U-Net backbone.

This channel attention mechanism is able to significantly enhance the salient features associated with lesions while suppressing the interference caused by background and staining differences. Under complex organisational structures, the SE module helps the model to better focus on key regions, thereby improving the robustness and recall of the segmentation.

#### 3.2.2. Organ-Aware Embedding Branch

**Figure 11:**
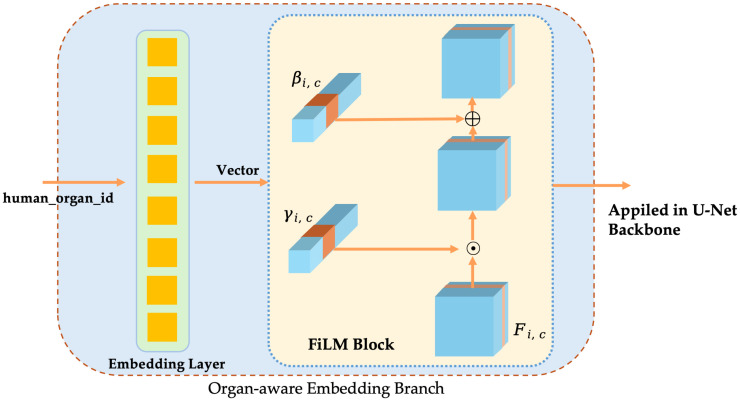
The Organ-aware embedding branch. Each organ name is mapped to a learnable embedding vector, which is transformed into FiLM parameters (γ,β) to perform channel-wise affine modulation on feature maps in the U-Net backbone.

Cross-organ/tissue pathological images have significant differences in morphology, textures, and staining, and a single model is easy to miss due to distribution transfer. In this study, organ-aware embedding is introduced, which enables the model to adaptively adjust the channel response across different organs by explicitly using organ labels to conditioning the features, thereby improving the recall, as shown in Figure 11.

1. Organ/tissue labels and embedding vectors
  Let the total number of organ categories be *K*. The organ labels y∈{1,…,K} of each sample are mapped to low-dimensional vectors by a learnable embedding layer:
  (1) $$z=\mathrm{Embedding}\left(y\right)\in R^{d}$$
  where *d* is the embedding dimension. This vector contains the category prior information of the organ/tissue.
2. FiLM conditional modulation
  At the *s*-th stage of the encoder, the graph is $F^{\left(s\right)}\in R^{B\times C_{s}\times H_{s}\times W_{s}}$. To inject conditional information, the embedding vector z is mapped through independent linear layers to a scaling factor $\gamma^{\left(s\right)}$ and a bias $\beta^{\left(s\right)}$ that is consistent with the number of channels:
  (2) $$\gamma^{\left(s\right)}=\sigma \;\left(W^{\left(s\right)}\gamma z+b^{\left(s\right)}\gamma \right),\;\beta^{\left(s\right)}=W^{\left(s\right)}\beta z+b^{\left(s\right)}\beta ,\;\gamma^{\left(s\right)},\beta^{\left(s\right)}\in R^{C_{s}}.$$
  where σ(·) is the Sigmoid activation used to constrain the scaling range. The feature maps are then obtained by a channel-wise affine transformation as follows:
  (3) $${\tilde{F}}^{\left(s\right)}=\left(1+\gamma^{\left(s\right)}\right)⊙F^{\left(s\right)}+\beta^{\left(s\right)}.$$
  This modulation process does not change the spatial dimension and the number of channels of the feature map, and recalibration is performed only in the channel dimension.
3. Multi-scale applications
  In order to inject conditional information in different receptive fields, FiLM is applied in the encoder and decoder stages ➀–➆ (corresponding channel $C_{s}$ = 64, 128, 256, 512, 256, 128, 64) (Shown in Figure 9). Separate linear layer parameters $W^{\left(s\right)}\gamma$ and $W^{\left(s\right)}\beta$ are used for each stage to ensure that the output dimension matches the number of channels.
  The computational and parameter overhead is given by $O\left(\sum_{s=1}^{4}C_{s}\cdot d\right)$. Compared to the backbone network, the overall computational complexity is negligible.

#### 3.2.3. Edge-Assisted Branch

In histopathology images, the boundary between tumour and normal tissue is often blurred, especially in the presence of inflammatory infiltration or tissue necrosis, so the segmentation model is prone to miss detection or inaccurate boundary. Therefore, this study introduces an edge-assisted branch in the last decoder layer of the U-Net backbone to enhance the sensitivity of the model to edge regions through explicit boundary supervision.

**Figure 12:**
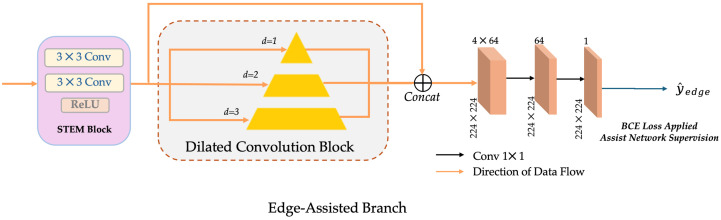
The Edge-assisted Branch. Decoder features before the final output are refined by a STEM block and multi-scale dilated convolutions (d=1,2,3), then projected into an edge map supervised by BCE loss with morphological-gradient ground truth.

High-resolution features $F_{\mathrm{dec}}\in R^{B\times C\times H\times W}$ from the decoder are first purified by STEM block (two layers of 3 × 3 convolutions + ReLU), and then fed into three parallel dilated convolutions with dilation rates of d = 1, 2, and 3, respectively to obtain multi-scale edge context. After concatenating the output of each branch, it is compressed into a single-channel edge prediction map ${\hat{Y}}_{\mathrm{edge}}$ by two layers of 1 × 1 convolution, as shown in Figure 12.

The edge labels are generated from the semantic segmentation mask via morphological gradient approximation, which is the difference between the dilation and erosion results. To maintain the differentiability of training, this study uses 2D Max pooling to approximate the dilation and erosion operations, resulting in a binary edge map as a supervision signal, which are shown in Equations (4) and (5):

(4) Dilate(X)≈MaxPool(X),Erode(X)≈1−MaxPool(1−X).

(5) $$Y_{\mathrm{edge}}\in {\left\{0,1\right\}}^{B\times 1\times H\times W}=\mathrm{Dilate}\left(X\right)-\mathrm{Erode}\left(X\right)$$

The edge prediction results are supervised by Binary Cross Entropy (BCE) loss with edge labels and jointly optimized with the segmentation main loss in the overall training. The introduction of this branch can guide the model to learn a clearer structure in the edge region so as to improve the segmentation accuracy of the lesion boundary while maintaining the overall recall rate.

In summary, this study introduces three complementary improved modules based on the classical U-Net to adapt to the characteristics of histopathological image segmentation. The SE block effectively enhanced the salient features related to lesions by modelling channel dependencies, while suppressing the interference caused by background and staining differences. The organ-aware embedding branch used the FiLM modulation mechanism to inject the prior information of the organ label into the encoder–decoder path so that the model can realise adaptive feature recalibration between different organs and tissues. The edge-aided branch guided the model to capture clearer structures in fuzzy boundary regions through explicit boundary supervision, thereby reducing false negatives at the lesion boundaries.

These three parts constituted an organ-aware and boundary enhanced segmentation framework, whose overall goal is to maximise recall while ensuring robustness, and better meet the clinical requirements for sensitivity and completeness. In the next section, the loss function design adopted in its training process will be introduced based on the network architecture.

### 3.3. Loss Functions

In histopathological image segmentation, a single loss function is difficult to balance recall and boundary accuracy at the same time.

- **Class imbalance problem:** The lesion area is often smaller than the background, and if only pixel-wise cross entropy (BCE) [52] is used, the model is easy to bias to predict the background, resulting in a decrease in recall.
- **Small lesion and fuzzy boundary problem:** If the model only relies on Tversky loss [53], although it can alleviate the imbalance, the constraint on the probability distribution is insufficient, and the training is unstable, especially in the small lesion area, so it is easy to oscillate.
- **Missing boundary problem:** The boundary between the lesion and normal tissue is fuzzy, and the main branch is not sensitive enough to the edge, which is easy to produce false negatives or broken boundaries.

Therefore, this study adopts a multi-loss joint design: the main segmentation branch is optimised by a novel composite loss, termed **HistoLoss**, while the edge-assisted branch is supervised by BCE loss to enhance boundary sensitivity in fuzzy regions.

#### 3.3.1. **HistoLoss**: A Recall-Prioritised Composite Loss for the Main Segmentation Branch

**HistoLoss** is designed as a weighted combination of Binary Cross Entropy (BCE) [52] and Tversky Loss [53] in order to ensure training stability while prioritising recall in pathological image segmentation.

BCE is a classical pixel-level loss function, which compares the predicted probability ${\hat{Y}}_{n}$ with the ground truth $Y_{n}$ for each pixel *n*:

(6) $$L_{\mathrm{BCE}}=-\frac{1}{N}\sum_{n}\left[Y_{n}\log{\hat{Y}}_{n}+\left(1-Y_{n}\right)\log\left(1-{\hat{Y}}_{n}\right)\right],$$

where *N* is the total number of pixels. BCE provides stable convergence by constraining the probability distribution.

Tversky Loss [53] generalises the Dice loss [54] by assigning asymmetric penalties to false positives (FP) and false negatives (FN), as shown below:

(7) $$L_{\mathrm{Tversky}}=1-\frac{\mathrm{TP}+\epsilon }{\mathrm{TP}+\alpha \cdot \mathrm{FP}+\beta \cdot \mathrm{FN}+\epsilon }.$$

In this study, α=0.3 and β=0.7 were used to increase the penalty on FN, thereby improving recall, which is critical in clinical diagnosis.

Finally, HistoLoss is defined as

(8) $$L_{\mathrm{Histo}}=0.3\;L_{\mathrm{BCE}}+0.7\;L_{\mathrm{Tversky}}$$

This design leverages the numerical stability of BCE and the recall-oriented nature of Tversky loss, ensuring both reliable optimisation and enhanced sensitivity to small lesions in histopathology segmentation.

#### 3.3.2. BCE Loss for Edge-Assisted Branch

In the edge-assisted branch, the model needs to learn to predict the binary image corresponding to the lesion boundary from the decoder features. Since the edge labels are binary and the difference between positive and negative samples is large (edge pixels only account for a small fraction of the total pixels), the BCE loss is chosen as the supervision function:

(9) $$L_{\mathrm{edge}}=-\frac{1}{N}\sum n\left[Y_{\mathrm{edge},n}\log{\hat{Y}}_{\mathrm{edge},n}+\left(1-Y_{\mathrm{edge},n}\right)\log\left(1-{\hat{Y}}_{\mathrm{edge},n}\right)\right]$$

where ${\hat{Y}}_{\mathrm{edge}}$ represents the predicted edge probability map, $Y_{\mathrm{edge}}\in \left\{0,1\right\}$ is the edge label generated by the segmentation mask by morphological gradient approximation, and *N* is the total number of pixels.

The advantage of BCE lies in its numerical stability and adaptability to sparse categories, which can provide reliable supervision signals in the case of unbalanced positive and negative samples, so that the model can still maintain a clear segmentation contour at fuzzy boundaries.

#### 3.3.3. Total Loss Function

Finally, the overall training objective is a weighted sum of the main segmentation loss and the edge-assisted loss:

(10) $$L_{\mathrm{total}}=w_{\mathrm{Histo}}\cdot L_{\mathrm{Histo}}+w_{\mathrm{edge}}\cdot L_{\mathrm{edge}}$$

where $w_{\mathrm{Histo}}$ and $w_{\mathrm{edge}}$ are the weights of the main segmentation branch and the edge helper branch, respectively. And $w_{\mathrm{Histo}}=1.0$ and $w_{\mathrm{edge}}=0.4$. This design ensures that the recall-optimised segmentation branch remains the primary supervision, while the edge branch provides auxiliary boundary refinement.

Overall, the design of loss function in this study takes into account three aspects: numerical stability, recall optimisation, and boundary refinement. The main branch strengthens the robustness to small lesions and class imbalance through the weighted combination of BCE and Tversky. The edge-assisted branch uses BCE to provide reliable supervision for sparse boundary pixels. The final total loss function integrates both in a weighted manner, thus striking a balance between overall segmentation accuracy and boundary continuity. The choice of loss weight in this study is based on the preliminary attempt on a small-scale validation set. It is found that it can steadily improve recall, so this combination is fixed in the subsequent experiments.

### 3.4. Human-in-the-Loop (HITL) Strategy

**Figure 13:**
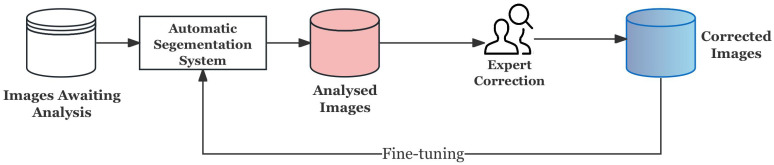
The overview of a standard HITL workflow.

In the workflow of clinical pathology, automatic segmentation systems usually do not directly replace experts, but serve as auxiliary tools, and their output still needs to be reviewed and corrected by pathologists if necessary. This human–machine collaboration mode is the core idea of Human-in-the-Loop (HITL) as shown in Figure 13, which can gradually improve the performance of the model on complex samples while ensuring clinical reliability.

However, building a complete online interactive HITL system involves user interface development, real-time model update, and feedback collection, which is beyond the scope of the work in this study. In order to verify the effectiveness of HITL while ensuring its feasibility, this study uses offline fine-tuning to simulate: That is, through the uncertainty-driven sample selection, the difficult examples are selected from a large number of prediction results instead of experts, and then these difficult examples are adjusted in a directional manner so as to approximate the process of “expert correction and feedback”.

#### 3.4.1. Why HITL?

- **Requirements for real scenarios:** Tumour metastatic lesions often have fuzzy boundaries and strong heterogeneity in morphology, which is prone to false negatives by simply relying on automatic segmentation models. But HITL allows for experts to supplement and correct key difficult cases to improve clinical reliability.
- **Limited annotation resources:** Medical image annotation is extremely expensive. HITL can maximise the value of limited annotation information by using a small number of expert corrections in the uncertainty sampling set.
- **Model interpretability:** The HITL framework not only outputs the segmentation results, but also provides the information of “which samples are the most uncertain” so that experts can understand the shortcomings of the model, thereby improving the transparency of human-machine collaboration.
- **Experimental feasibility:** Compared to building an online system, the offline fine-tuning scheme is simpler to implement, but it can effectively simulate the HITL idea at the experimental level and quantitatively evaluate its impact on model performance.

#### 3.4.2. Offline HITL for Proposed Architecture

**Figure 14:**
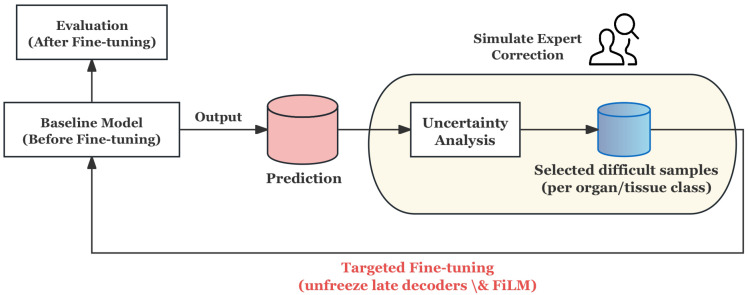
The HITL mechanism utilised in this study.

The specific process is shown in Figure 14, which is mainly divided into the following steps:

1. **Prediction and Uncertainty Evaluation:** The baseline model generates probability maps for training samples, and the prediction uncertainty is quantified by calculating the entropy of the probability distribution, thereby identifying the samples with the lowest model confidence.
2. **Stratified Uncertainty Sampling:** The most uncertain samples in each organ category are selected to form the HITL subset. This ensures that difficult cases in each category are covered and that approximate simulation experts give priority to ambiguous or error-prone cases in the clinic.
3. **Targeted Fine-tuning:** Instead of updating the whole model, only the decoder and its conditioning module are unfrozen for refinement, and the rest of the network remains frozen. This design can reduce the risk of overfitting and enable the model to focus corrections on segmentation boundaries and organ-specific features.
4. **Evaluation and Feedback Loop:** After fine-tuning on the HITL subset, the model is evaluated on an independent test set. The process simulates the feedback loop of “prediction → expert correction → model update”, which proves that the model can improve the segmentation reliability in fuzzy boundary and heterogeneous regions, even under the condition of limited correction.

Without building a real-time system, this offline HITL strategy shows the closed-loop mechanism of “model candidates → expert identification of difficult cases → expert correction feedback → model improvement” in clinical scenarios. The experimental results show that the uncertainty-driven hierarchical sample selection and small-scale directional fine-tuning can effectively absorb the information corrected by experts so as to improve the segmentation performance in regions with fuzzy boundaries and tissue heterogeneity of metastases.

### 3.5. Recall-Oriented Design Rationale

ClinSegNet was developed with a recall-oriented philosophy, where each architectural component is designed to mitigate a specific type of false negative (FN):

- **SEU-Net** serves as the fundamental architecture. The U-shaped encoder–decoder captures multi-scale context, while SE (Squeeze-and-Excitation) recalibrates channel responses. This combination enhances sensitivity to subtle lesion patterns and reduces FNs in heterogeneous tissue regions.
- **HistoLoss (main branch)** addresses FNs from small lesions and long-tail positive samples that are often overwhelmed by the background. As a combination of BCE and Tversky with β>α, HistoLoss penalises false negatives more strongly, while the BCE term ensures training stability. This loss encourages the model to prioritise sensitivity to positive regions.
- **$L_{\mathrm{edge}}$ (edge supervision)** alleviates FNs caused by broken or blurred boundaries. The auxiliary edge prediction head is trained with a BCE-based loss, which provides explicit contour supervision and reduces the likelihood of missing boundary pixels.
- **Conditional guidance (FiLM + organ embeddings)** mitigates FNs due to cross-organ morphological variation. By injecting organ embeddings through FiLM layers and recalibrating channels with SE blocks, the network adapts to organ-specific structures and reduces domain-shift-induced FNs.
- **Colour processing** handles FNs introduced by staining variability across tissues or batches. By suppressing colour and emphasising morphological cues, the model becomes less sensitive to stain shifts that obscure positive regions.
- **Offline HITL (uncertainty-driven fine-tuning)** targets FNs in difficult cases that persist after initial training. By selecting high-uncertainty samples and fine-tuning only the decoder’s final layers and conditional embeddings, the system converts critical FNs into true positives at low cost.

Together, these components form ClinSegNet as a recall-oriented segmentation framework, where each module directly addresses a different type of FN. While this design inevitably sacrifices part of Dice and IoU, it achieves the primary goal of reducing missed detections in clinical pre-screening scenarios.

## 4. Experimental Settings

### 4.1. Environment Settings

Due to the training process of deep learning models is highly dependent on computing resources, and differences in experimental environments may affect model convergence and performance, before formally describing the training and fine-tuning strategy, we first explain the adopted hardware and software configuration in Table 5 to ensure the transparency and reproducibility of the experiment.

### 4.2. Main Model Training Settings

During the training stage, all images and their corresponding masks are uniformly scaled to a resolution of 224×224. Bilinear interpolation is used for image scaling, and nearest-neighbour interpolation is used for mask scaling to avoid blurring the segmentation boundaries. Subsequently, the data is converted into tensor form and fed into the network.

The model utilises the proposed U-Net structure of conditional embedding (Shown in Figure 9). The input is a single-channel pathological image, and the corresponding embedding vector is generated by combining the organ label. The embedding dimension is set to 32 to realize the modulation of different organ features. The batch size is set to 8 and the maximum number of iteration rounds is 50.

Adam [55] is selected as the optimiser, and the learning rate is set to $5\times 10^{-4}$. In the experiment, the training set and the test set are strictly divided, and the organ category is uniformly encoded by the mapping dictionary organ_to_label, which ensures the consistency of the conditional embedding.

### 4.3. HITL Settings

In the HITL stage, based on the entropy distribution predicted by the model, this study first selects high entropy samples in each category to form a manual correction subset, and the ratio is selected as hitl_ratio (default 0.30). Each class should have amount min_per_class (default 2) at least.

For targeted fine-tuning, the strategy is that model only unfreeze the last two decoder stages (➅ and ➆, shown in Figure 9), the corresponding FiLM condition module and the output layer, and keep the other parameters frozen.

**Figure 15:**
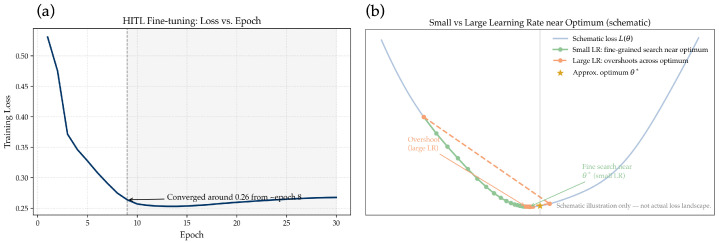
(**a**) Training loss during HITL fine-tuning, showing rapid convergence around epoch 8. (**b**) Schematic illustration of learning rate effects: small LR enables fine search near the optimum, while large LR overshoots and oscillates. (For illustration only, not an actual loss landscape).

To empirically validate the choice of hyperparameters in the HITL fine-tuning phase, additional analyses were conducted on a small validation subset. As shown in Figure 15a, the training loss rapidly decreases and converges around 0.26 after approximately eight epochs, indicating that the model reaches a stable optimum without further benefit from additional iterations. Extending the training beyond this point slightly increases the loss, suggesting the onset of overfitting. Therefore, the fine-tuning stage was limited to eight epochs to maintain stability and efficiency.

Regarding the learning rate, Figure 15b schematically illustrates the rationale behind using a smaller value ($2\times 10^{-4}$). In the HITL stage, the model operates near an existing optimum derived from the main training. A small learning rate enables fine-grained exploration within this region, whereas a large learning rate tends to overshoot the optimum, potentially disrupting the previously learned feature representations. Hence, a conservative learning rate was adopted to achieve stable convergence and precise adjustment of the decoder layers.

Using Adam optimiser [55] on the HITL subset (learning rate $2\times 10^{-4}$, weight decay $10^{-2}$), batch size 8, training for eight epochs, OneCycleLR learning rate scheduling, Automatic Mixed Precision (AMP) [56], and Exponential Moving Average (EMA) [57] are combined to improve the training stability and generalization ability. And the loss function design is kept consistent with the main training stage.

### 4.4. Evaluation Metrics

In the experiment, the primary evaluation index is recall, and its core objective is to minimise the missed detection of the lesion area as much as possible. In addition, the Dice coefficient, Intersection over Union (IoU), and precision are combined as supplementary indicators for common segmentation tasks to comprehensively evaluate the performance of the model. These metrics are defined based on the confusion matrix shown in Table 6.

Recall reflects the sensitivity of the model detection, and is defined as follows:
  (11) $$\mathrm{Recall}=\frac{TP}{TP+FN}.$$
The *Dice* coefficient is used to evaluate the overlap between the prediction and the true mask, and the equation is as follows:
  (12) $$\mathrm{Dice}=\frac{2TP}{2TP+FP+FN}.$$
Intersection over Union (*IoU*) measures the ratio of the intersection and union between the predicted region and the true region, and is defined as follows:
  (13) $$\mathrm{IoU}=\frac{TP}{TP+FP+FN}.$$
*Precision* measures the reliability of the positive samples in the prediction, as follows:
  (14) $$\mathrm{Precision}=\frac{TP}{TP+FP}.$$

*In* the training stage, these metrics are used to monitor the performance of the model on the validation set, where *recall* is used as the main criterion to preserve the best model to minimise the miss rate. *Dice*, *IoU*, and *precision* are commonly used as complementary metrics to help analyse the overall level of segmentation quality. In the HITL stage, the manually corrected samples are also re-evaluated based on the above indicators to verify the improvement effect of fine-tuning on *recall* and overall segmentation performance. All experiments were conducted in a unified environment, and *recall* was used as the main evaluation criterion, which is comprehensively evaluated by combining *Dice*, *IoU*, and *precision*. Based on the above method design, the experimental results are presented and analysed in the next section to verify the effectiveness of the proposed framework in terms of *recall* improvement and margin optimisation.

## 5. Results

To systematically evaluate the effectiveness and applicability of the proposed method, this study conducted experiments on two different datasets. As shown in Figure 8, firstly, baseline training and HITL simulation were carried out on the publicly available NuInsSeg dataset to verify the feasibility and robustness of the framework in multi-organ/tissue scenarios. And then the UHS-MelHist dataset was combined with NuInsSeg to construct a larger-scale and more clinically relevant NuInsSeg-UHS-Combined dataset and complete training and HITL experiments. To ensure the representativeness of the results, ablation experiment and comparison experiments were all conducted on the combined dataset, which verified the contributions of each module and the overall performance advantages under more complex data distributions.

### 5.1. Quantitative Results

#### 5.1.1. NuInsSeg: Training vs. HITL

Table 7 presents the baseline training results and the fine-tuning results with HITL on the NuInsSeg dataset. It can be observed that the recall increased from 0.8803 to 0.8983 after HITL, with an increase of approximately 2.05%. It demonstrated the role of the human–computer interaction process in reducing false negatives. Since this study takes recall as the core evaluation criterion, the baseline stage only reports this metric, while precision, Dice, and IoU are presented only in the HITL stage as supplementary explanations. Overall, this result verifies the effectiveness of the proposed method on the public dataset.

Based on the current result, to further evaluate the performance of the method in more complex scenarios, the experiment continued to be conducted on the constructed NuInsSeg-UHS-Combined dataset. This dataset had a larger scale data distribution closer to the real clinical applications so that it can better reflect the generalisation ability of the model in practical applications. Next, the results under baseline training and HITL fine-tuning will be presented, respectively.

#### 5.1.2. NuInsSeg-UHS-Combined: Training vs. HITL

Table 8 shows the results of the NuInsSeg-UHS-Combined dataset. It can be observed that the recall after HITL increased from 0.8917 to 0.9053. It is an increase of 1.53% which reduces the missed detection in images. Different from the public dataset, precision, Dice and IoU are also significantly improved on this large-scale dataset, increasing by 12.96%, 9.02% and 11.53% respectively.

This further shows that HITL can improve the sensitivity and also improve the overall quality and boundary consistency of the prediction results in more complex clinical scenarios. Moreover, it also reflects the value of proposed method in practical applications.

#### 5.1.3. Comparison Experiment

On the NuInsSeg-UHS-Combined dataset, this study further conducts a horizontal comparison with several representative segmentation models to verify the rationality and performance level of the proposed method. The selected baselines cover different categories of mainstream methods:

- U-Net [14] and Attention U-Net [16] represents the classical Convolutional Neural Network (CNN) architecture, which is widely used in medical image segmentation tasks.
- SwinU-Net [21] and TransUNet [17] combine Transformer and U-Net, reflecting the recent trend of the fusion of vision Transformer and convolution.
- DeepLabV3+ [29] is a strong baseline commonly used in the field of semantic segmentation. It can effectively model context by using dilated convolution and multi-scale feature aggregation.
- MedT [20] represents a pure Transformer architecture, which is useful for medical imaging tasks.

By comparing in these representative frameworks, the relative performance of this research method under different types of networks can be comprehensively investigated, and thus its effectiveness under the high-recall target can be more strongly illustrated. Table 9 shows the horizontal comparison results on the NuInsSeg-UHS-Combined dataset.

Despite adding FiLM, SE, and an edge-assist branch, the overall parameter count of ClinSegNet remains compact. This is because the extra modules are channel-wise and lightweight: FiLM adds only $O(\sum_{s}C_{s}\cdot d)$ linear mappings (with d = 32), SE adds two small fully connected layers per stage, far below convolutional $O(C_{s}^{2}k^{2}))$, and the edge head reuses decoder features with a shallow 1 × 1 projection. As a result, most weights still reside in the backbone convolutions, keeping Params = 9.79 M even though the architecture is richer. The GFLOPs (≈84)remain close to classical U-Net because FLOPs are dominated by backbone convolutions on 224 × 224 patches, while FiLM and SE introduce negligible additional compute overhead.

Overall, the comparative analysis reveals clear differences in both segmentation performance and computational efficiency among the tested models. Traditional convolutional networks such as U-Net and Attention U-Net exhibit balanced Dice and IoU scores with moderate complexity, but their limited recall leads to higher false negative rates. Transformer-based methods such as SwinU-Net and TransU-Net further increase computational cost while yielding only marginal improvements in recall, indicating limited efficiency-to-performance benefit.

In contrast, ClinSegNet maintains a compact structure with 9.79 M parameters and 83.9 GFLOPs, comparable to CNN-based baselines, while achieving a recall of 0.8917—second only to MedT. Its average inference latency of 1.38 ms per image also demonstrates competitive efficiency, suggesting strong potential for real-time or large-scale histopathology screening.

**False Negative Reduction (FNR):** Given that recall (*R*) is defined as shown in Equation (11),

(15) $$R=\frac{TP}{TP+FN}\Rightarrow FN=TP\left(\frac{1}{R}-1\right).$$

The relative false negative reduction rate can be derived by assuming an equal number of true positives (TP) across models:

(16) $$\mathrm{FNReduction}=\frac{FN_{\mathrm{baseline}}-FN_{\mathrm{ours}}}{FN_{\mathrm{baseline}}}=1-\frac{FN_{\mathrm{ours}}}{FN_{\mathrm{baseline}}}=1-\frac{\left(\frac{1}{R_{\mathrm{ours}}}-1\right)}{\left(\frac{1}{R_{\mathrm{baseline}}}-1\right)}.$$

Positive values indicate that ClinSegNet yields fewer missed detections than the baseline, while negative values correspond to higher recall in the baseline model (e.g., MedT). The results in Table 9 show consistent FN reduction compared to the CNN and hybrid Transformer baselines, confirming the model’s advantage in recall-oriented sensitivity enhancement. This reduction in false negatives directly supports the clinical motivation of minimizing missed metastatic regions while maintaining efficient computation.

It can be seen from the results that some traditional convolutional networks (such as U-Net and Attention U-Net) have relatively balanced performance in comprehensive indicators such as Dice and IoU, and MedT has the highest value in recall, but the precision is very low, and the overall segmentation quality is limited. The proposed method in this study achieves 0.8917 in recall, second only to MedT, while maintaining the level of mainstream models, but relatively low in precision, Dice, and IoU. This result is in line with the design goal of this study, indicating that the model has advantages in reducing missed detection.

#### 5.1.4. Ablation Study

To verify the effectiveness of different components in the proposed framework, ablation experiments were performed in this study. By gradually removing SE modules, FiLM conditional embedding, and Edge-assisted supervision, the specific contribution of each component in the overall performance improvement can be clarified, so as to verify the rationality of the complete model design. Table 10 shows the ablation experimental results on the NuInsSeg-UHS-Combined dataset.

It can be seen that the performance of the model decreases to varying degrees after removing some modules, indicating that all components play a positive role in the overall framework. Specifically, both Dice and IoU decrease when removing the SE module, indicating that the enhancement of channel features by SE contributes to the overall segmentation quality. The relative decrease of recall when removing FiLM indicates that organ conditional embedding plays a role in ensuring sensitivity. When the Edge auxiliary supervision is removed, the boundary related indicators (IoU and Dice) are significantly reduced, indicating that edge branch is effective in improving boundary consistency. Overall, the complete model integrating SE, FiLM, and Edge modules achieves 0.8917 in recall, which reflects the advantage in reducing missed detection. Although it is not dominant in Dice and IoU, this result is in line with the design idea of this study with high recall as the priority. Furthermore, the complementary statistical analysis revealed that the paired *t*-test was less sensitive under non-normal data distributions, whereas the Wilcoxon signed-rank test more accurately captured the consistent performance gains of each module, confirming their practical contribution to the overall framework.

#### 5.1.5. Per-Organ Recall Analysis

Table 11 shows the comparison results of organ-by-organ recall on NuInsSeg and NuInsSeg-UHS-Combined datasets.

Overall, with the introduction of UHS samples (referred to as **Melanoma LN**), the recall of most organ categories shows a noticeable improvement, indicating that the model’s lesion coverage becomes more consistent when the data distribution is expanded towards real clinical conditions. This improvement suggests that incorporating clinically derived cases helps the model generalise better to heterogeneous tissue patterns and staining variations, thereby enhancing its robustness and reliability in detecting metastases under diverse pathological appearances.

**Figure 16:**
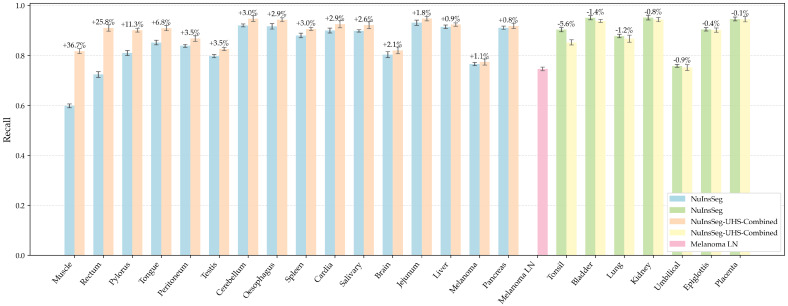
Per-organ recall analysis between NuInsSeg and NuInsSeg-UHS-Combined datasets. Most organs show improved recall after UHS integration, with Muscle, Rectum, and Pylorus gaining the most, while Melanoma LN appears only in the combined dataset.

Among them, **Rectum (0.7236 → 0.9102)** and **Muscle (0.5981 → 0.8175)** have the most significant increase as shown in Figure 16, which indicated that UHS samples provide additional effective information for complex tissue boundaries and difficult to segment regions. At the same time, some organs such as Umbilical and Tonsil fluctuated slightly in recall, but the total recall is still maintained in a high range. Overall, these results are consistent with the qualitative analysis, which further validated the universality of the proposed method in multi-organ scenarios and the enhanced recall of difficult categories after fusing clinical data.

#### 5.1.6. Hyperparameter Sensitivity Analysis

To further validate the robustness of model design choices, this subsection analyses the sensitivity of key hyperparameters, including the FiLM embedding dimension and the **HistoLoss** weighting ratio.

Regarding the FiLM embedding size (Table 12), the model achieves the highest recall (0.8948) at a dimension of 64, although with greater variance (SD = 0.0227). A smaller dimension of 32 provides a slightly lower recall (0.8879), but demonstrates exceptional stability (SD = 0.0004) and achieves the best Dice and IoU scores (0.7763 and 0.6517, respectively). In contrast, larger dimensions (≥128) yield diminishing returns and even reduced performance, indicating that the embedding capacity saturates around 64.

**Figure 17:**
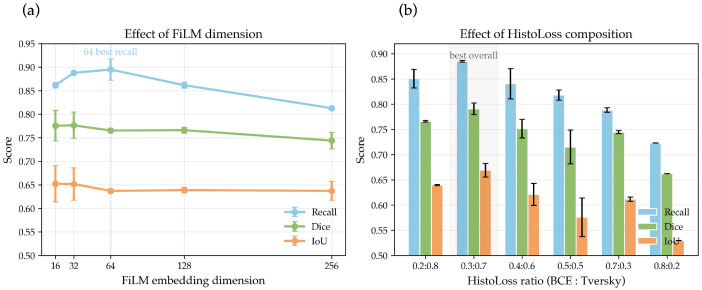
Hyperparameter sensitivity analysis. (**a**) Effect of FiLM embedding dimension on recall, Dice, and IoU (mean ± SD). (**b**) Effect of HistoLoss ratio (BCE:Tversky), where the 0.3:0.7 balance achieves the best overall performance.

As shown in Table 13, the composite HistoLoss achieves the best trade-off when the BCE:Tversky ratio is set to 0.3:0.7, yielding the highest recall (0.8849) and the most stable overall segmentation quality. A lower Tversky weight (e.g., 0.5:0.5) leads to unstable optimisation, as the loss becomes dominated by pixel-wise cross-entropy, which tends to bias the model towards background regions and ignore hard-to-detect minority pixels. Conversely, a higher Tversky dominance (e.g., 0.2:0.8) improves recall by emphasising false negative suppression, but slightly degrades boundary consistency, as excessive weighting toward positive samples can weaken contour precision. The 0.3:0.7 configuration provides an effective balance, maintaining strong sensitivity to small or faint lesions while preserving clear boundaries and overall Dice performance. This observation is further supported by the visual comparisons in Figure 17, where the 0.3:0.7 setup produces the most complete and anatomically consistent segmentation contours across different organ categories.

Overall, these results suggest that ClinSegNet maintains robust segmentation performance within a lightweight parameter range (9.7 M–10.4 M), and that both the balanced 0.3:0.7 HistoLoss configuration and moderate FiLM embeddings (32–64 dimensions) effectively enhance recall and generalisation stability without introducing substantial computational overhead. The combination of these settings enables the model to focus on hard-to-detect lesion regions while avoiding over-sensitivity to noise, thereby improving sensitivity in a controlled manner. This balance between structural simplicity and functional adaptability allows for ClinSegNet to achieve recall-oriented optimisation with minimal trade-offs in efficiency, providing a practical foundation for real-world pathological segmentation systems where computational resources and diagnostic precision must be jointly considered.

### 5.2. Qualitative Results

In order to further demonstrate the intuitive effect of the proposed method, this section conducts qualitative analysis from two perspectives: First, it compares the segmentation results of different models on multiple organ samples and the correction effects before and after HITL. And the second one is to visualise the response and uncertainty distribution of the model to the lesion area through the heat map.

#### 5.2.1. Segmentation Results Visualisation

**Figure 18:**
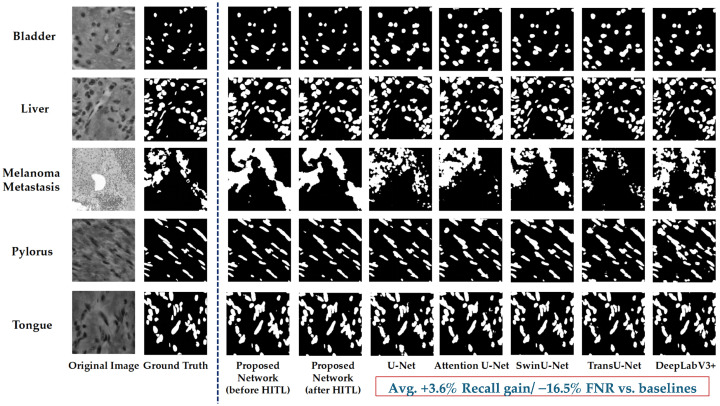
Qualitative comparison of multiple organs, including bladder, liver, Melanoma Metastasis, pylorus, and tongue. From left to right: Original image, Ground Truth, Proposed Network (before HITL), Proposed Network (after HITL), U-Net, Attention U-Net, Swin U-Net, TransUNet, and DeepLabV3+.

Figure 18 shows the results for multiple organs. In general, the results of different models in bladder, liver, pylorus, tongue, and other tissues are not significantly different, and the overall segmentation results covered the main lesion area well. However, in the Melanoma Metastasis scenario, the difference is particularly prominent: CNN methods such as U-Net and Attention U-Net were prone to obvious missed detection when dealing with scattered small lesions, resulting in insufficient recall. Transformer methods such as SwinU-Net and TransU-Net could maintain a certain boundary shape in the general area, but the prediction was unstable at the edge of complex lesions, often resulting in over-expansion or boundary fracture. DeepLabV3+ performed moderately well in some continuous regions, but fragmented predictions still occurred in regions with complex structures or dense lesions. In contrast, the proposed method has more advantages in the recall and boundary integrity of small lesions, can cover the scattered metastatic lesions more completely, and the prediction results are closer to the real annotation as a whole. After HITL fine-tuning, the model repaired part of the missed regions and reduced the redundant noise in the prediction, which made the segmentation results clearer. It further highlighted the applicability and robustness of the proposed method on clinical high-risk samples.

**Figure 19:**
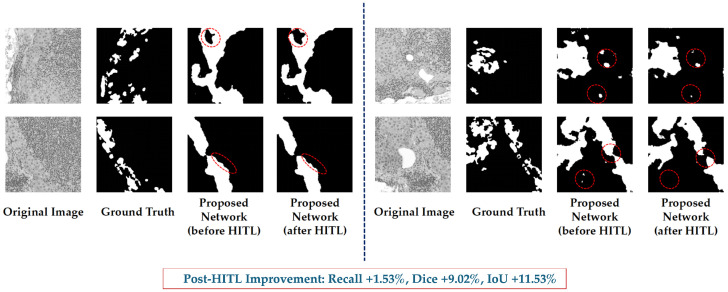
Effect of HITL fine-tuning on melanoma metastasis cases. From left to right: Original image, Ground Truth, Proposed Network (before HITL), Proposed Network (after HITL).

To further explore the HITL mechanism on real clinical data, the Figure 19 shows the effect of HITL on the UHS-MelHist dataset.

It can be observed that although the model prediction covers the main lesions, there are more noisy regions and the boundary contour is not accurate enough before HITL. After the fine-tuning of HITL, the noise in the prediction results is significantly reduced, and the boundaries are more consistent with the real labels so that the overall segmentation results are cleaner and more stable. The area marked by the red circle intuitively reflects this improvement, which indicated that HITL mechanism improved the recall rate and also effectively increased the boundary consistency and segmentation reliability in clinical source samples.

#### 5.2.2. Heatmap Visualisation

In order to more intuitively show the response of the model in the lesion area, this study further draws the prediction probability heatmap.

**Figure 20:**
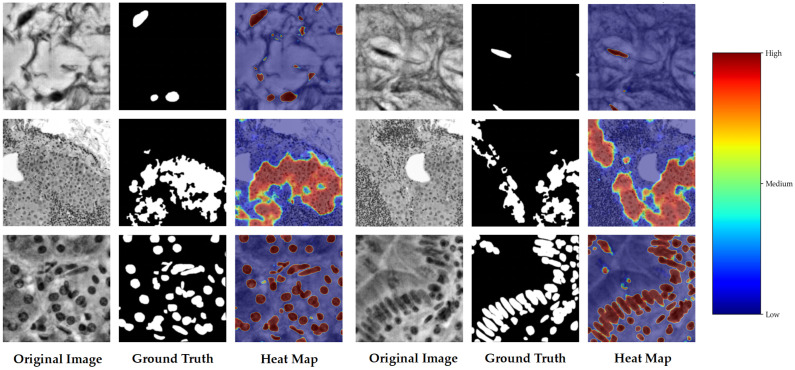
Heatmap visualisation of model predictions. The colour intensity indicates the probability of lesion prediction, with warmer colours representing higher confidence.

The heatmap overlays the probability distribution of the model output on the original greyscale image, which can intuitively reflect the attention degree of the model to the lesion area. As shown in Figure 20, the model showed an obvious highlighting response in the real lesion area, while the response in the normal tissue area was weak, indicating that the segmentation results of the model were indeed based on the effective learning of lesion features, rather than chance or spurious correlation. This visualization provides intuitive support for the quantitative indicators and also further verifies the reliability of the proposed method in identifying the lesion area. More detailed analysis and implications will be discussed in the following Discussion section.

## 6. Discussion

### 6.1. Overall Performance

The experimental results show that the proposed framework had excellent overall performance in the multi-organ pathological image segmentation task, especially in recall. The reason was not only the introduction of a single module, but also the complementarity and cooperation of multiple mechanisms. The SE module obtained channel attention weights through global average pooling, which could strengthen the fine-grained features related to lesions in the background-dominated image, so as to avoid small lesions being submerged in the downsampling process. FiLM conditional embedding learned the low-dimensional representation of organ labels and used scaling and translation parameters to dynamically modulate the distribution of different tissues at the feature level so that the model could “discriminate” different organs and effectively reduce cross-organ confusion. By introducing additional boundary loss into the prediction branch, the edge-assisted supervision made the network maintain higher sensitivity to the transition region between the lesion and the background during training, reducing the risk of boundary breakage. The synergy of the three made the model not only maintain global sensitivity, but also ensure discrimination in local boundaries and cross-organ scenes, thus significantly improving recall.

### 6.2. Effectiveness of HITL Mechanism

The introduction of HITL mechanism further guaranteed improvements in recall. With limited computational cost, the entropy value was used as the uncertainty measure. Specifically, the samples with the least confidence of the model are preferentially included in the fine-tuning subset, and each organ category was guaranteed to contain at least several difficult samples. Compared with random sampling, this strategy could be closer to the clinical needs: The model focused on the high-risk regions with fuzzy boundaries and strong heterogeneity, so as to bring higher recall gain at the same cost. Fine-tuning only on these samples was equivalent to directional reinforcement of the model on the weakest link, which not only avoided overfitting the full model, but also significantly reduced the risk of missed detection. In terms of parameter update mode, HITL only unfroze the last two layers of the decoder and the related modulation modules. This local plasticity strategy enabled the model to quickly adapt to difficult examples without destroying the original global representation. Because of this, HITL showed a steady improvement in recall in our experiments, accompanied by improvements in boundary fit and noise level. It could be seen that HITL was not a simple data supplement, but a mechanism that matched with the model structure and played a role specifically for the recall optimisation goal.

It should be noted that the improvement in overall recall brought by HITL is not very significant. However, in the task of pathological image segmentation, the recall of baseline (before HITL) was already close to 0.89, which is at a high level. In this case, even a 1–2% improvement is valuable. If the purpose of applying HITL is that making the model concentrates on difficult examples, this increase exactly reflected the effectiveness of HITL. In other words, HITL did not aim for a large increase in overall performance, but rather reduced missed detections in the most error-prone cases through targeted fine-tuning, thus making it more practical in clinical practice.

Therefore, the HITL setting in this study should be understood as an offline uncertainty-guided fine-tuning scheme that aims to verify the potential value of the HITL idea in the pathological segmentation scenario, rather than building a complete interactive platform.

Nevertheless, a clear limitation of the current HITL design is that the process was simulated offline without direct participation of pathologists. As such, the present findings primarily demonstrate the potential of uncertainty-based sample selection rather than the full effectiveness of real human–AI collaboration. In real clinical workflows, pathologists may provide nuanced feedback that reflects contextual interpretation, visual reasoning, and diagnostic experience, which cannot be fully replicated in an offline simulation. These human factors, including subjectivity, decision latency, and interaction habits, may have non-negligible impacts on model adaptation and usability outcomes.

In future work, a prototype interactive platform could be developed to integrate expert feedback directly into the inference loop. For instance, uncertain or misclassified regions could be dynamically highlighted and reviewed by pathologists, whose annotations or accept/reject actions would be used to iteratively refine the model parameters. Such a design would not only enable quantitative assessment of user efficiency, interaction latency, and diagnostic reliability, but also help to explore how the model and human experts can mutually adapt during continuous learning. In this way, the clinical feasibility and robustness of the proposed HITL framework could be further validated in real-world diagnostic environments, bridging the current gap between simulation and actual pathology practice.

### 6.3. Comparison with Representative Baseline Models

The horizontal comparison experiments revealed the characteristics and limitations of different types of methods in recall and other indicators. Classical convolutional networks (such as U-Net [14] and its attention variant [16]) performed relatively balanced on Dice and IoU, indicating that their predictions were stable in continuous regions, but they relied on local receptive fields and were difficult to cover small lesions with scattered distribution, resulting in always low recall. These methods were easy to miss detection in pathological image segmentation, which limited the clinical application value.

Hybrid architectures incorporating transformers, such as SwinU-Net [21] and TransU-Net [17] improved global modelling capabilities to some extent, but this capability did not translate into significant recall gains. SwinU-Net was stable in boundary preservation, but it was still not sensitive to small lesions. However, TransU-Net was prone to boundary breakage or over-expansion in complex areas, indicating that its global modelling advantages had not yet fully adapted to the needs of high heterogeneity and fine granularity in pathological images.

Although DeepLabV3+ [29] performed well in natural image segmentation, its recall was low in pathological scenes, reflecting that its dilated convolution and multi-scale context aggregation mechanism could not meet the challenge of lesion recognition well, especially in small lesions.

A quantitative comparison further supports this observation. As shown in Table 9, MedT attains a recall of 0.9562, but a precision of only 0.4291, whereas ClinSegNet reaches a recall of 0.8917 with a much higher precision of 0.6869. This corresponds to roughly a 35–40% reduction in false positives while maintaining high sensitivity, suggesting that ClinSegNet achieves a more clinically balanced trade-off between sensitivity and specificity. Such a balance is critical for practical deployment, as excessive false positives can increase pathologists’ workload, whereas ClinSegNet’s more stable predictions align better with the efficiency and reliability required in diagnostic screening.

In this context, the proposed method in this study was second only to MedT in recall, but maintained the mainstream level in precision, Dice, and IoU, achieving a more balanced performance. Compared to other convolution or hybrid methods, the proposed method had obvious advantages in recall. Compared to MedT, the stability of prediction quality was gained by sacrificing a small amount of recall, and the false positive was avoided. This was highly consistent with the design goal of this study: to maintain the usability of segmentation results while improving recall as much as possible, thus effectively reducing the risk of missed detection in clinical diagnosis.

### 6.4. Dataset Constraints and Generalisation Potential

A limitation of this study is that the UHS-MelHist dataset is relatively small and derived from a single clinical source, which limits the clinical diversity and constrains direct evidence of cross-hospital generalisation. Although ClinSegNet has demonstrated robust segmentation performance within this distribution, further evaluation is needed to verify its adaptability to unseen data from different scanners, staining protocols, or pathological contexts. The combination with the public NuInsSeg dataset was therefore intended not only to augment sample size, but also to introduce greater variability in tissue morphology and staining appearance, thereby improving the model’s cross-domain robustness.

The proposed architecture was deliberately designed to enhance transferability through its structural components: FiLM embeddings enable organ-aware contextual adaptation, while SE blocks and edge-assisted supervision strengthen resilience to staining inconsistency and boundary ambiguity. These mechanisms suggest that ClinSegNet could be extended to other melanoma metastasis sites or even to distinct pathological tissues beyond melanoma, provided adequate fine-tuning or domain-specific calibration.

In future work, we plan to collaborate with additional clinical teams to acquire larger and more diverse cohorts encompassing multiple organs and staining variations. The ultimate goal is to gradually replace the mixed-source dataset with a fully clinical corpus, enabling comprehensive validation of the proposed framework’s generalisation ability and its reliability in real-world diagnostic workflows.

### 6.5. Clinical Relevance

In histopathological diagnosis, the consequences of missed detection are far more serious than those of false detection. False detection usually requires only further verification by the pathologist to rule it out, whereas missed detection means that a lesion is completely ignored, directly affecting diagnostic integrity and potentially endangering patient safety. Therefore, the inclusion of recall as the main optimisation objective in this study is of direct clinical interest in itself.

It should also be noted that the improvement in recall was accompanied by a slight reduction in precision, reflecting a deliberate trade-off between sensitivity and specificity. From discussions with our collaborating histopathologist co-authors at University Hospital Southampton, it was emphasised that in histopathology screening, higher recall with moderate over-segmentation is clinically preferable to the risk of missed metastases. This viewpoint reflects a widely accepted diagnostic principle that prioritises sensitivity in early-detection tasks, where false negatives are considered more detrimental than false positives. The feedback from our clinical co-authors aligns with the established screening literature, where recall-oriented systems are regarded as safer for initial triage and screening [58,59,60]. Consequently, the recall-oriented design of ClinSegNet represents a clinically informed balance between sensitivity and workload rather than a reduction in diagnostic reliability.

This was further demonstrated by the introduction of HITL. By focusing fine-tuning on the samples where the model was most uncertain and error-prone, the model was directly strengthened at these critical links. Such improvements did not seek to enhance all metrics uniformly, but rather reduced the risk of missed detection in the most error-prone cases. Although this strategy yielded limited quantitative improvements, it was more consistent with the actual needs of clinical applications.

More importantly, this demonstrated the value of the human–machine collaboration paradigm. Instead of replacing an expert, the model was constantly refined through the expert calibration process, thereby enhancing sensitivity to critical lesions while ensuring stability. This framework pointed to a more forward-looking application scenario: the segmentation system not only aimed for high recall, but also continuously improved through iterative interaction to approximate the real diagnostic process.

In terms of practical integration, ClinSegNet could be embedded into the digital pathology workflow as a pre-screening and triage tool. Its high-recall segmentation outputs could highlight suspicious metastatic regions on whole-slide previews, allowing for pathologists to focus on high-risk areas first and reduce manual search time. The edge-assisted branch can further provide boundary cues during annotation, assisting in faster and more consistent delineation of tumour margins. Meanwhile, the uncertainty-guided HITL mechanism offers a feasible route for real-time human–AI interaction, where pathologists review uncertain areas and feed corrections back to the system for continual optimisation. Together, these functions illustrate how ClinSegNet could serve as a supportive decision-assistive module that enhances diagnostic efficiency while preserving clinical oversight.

## 7. Conclusions

This study proposed ClinSegNet, a recall-oriented segmentation framework integrating organ-aware embeddings, edge-aided supervision, and uncertainty-guided fine-tuning. Experiments on the NuInsSeg and UHS datasets demonstrated consistent improvements in recall and boundary coherence across diverse histopathological conditions, confirming the framework’s reliability and clinical relevance.

Despite its promising results, this study has several limitations. The scale of the UHS dataset remains limited, and the combined use with public data, though helpful for training stability, may constrain clinical generalisability. Moreover, while ClinSegNet maintained strong recall, moderate trade-offs in precision and contour accuracy suggest room for further refinement of segmentation quality.

It is also acknowledged that ClinSegNet did not consistently outperform all baselines in Dice and IoU. This reflects an inherent trade-off of recall-oriented design: reducing false negatives may come at the cost of slightly higher false positives or reduced contour precision. Nevertheless, the stability of recall achieved by ClinSegNet aligns with the intended clinical objective of reducing missed detections. It is also noted that the HITL mechanism was only applied to ClinSegNet. While the results demonstrated its effectiveness within the proposed framework, further work is needed to apply the same strategy to baseline models in order to verify its generality.

Moreover, several technical limitations should be noted. First, the input resolution was restricted to 224×224, which may lead to missing very small lesions; higher resolutions or multi-scale strategies could alleviate this risk. Second, all images were converted to greyscale to mitigate staining variability, which improved cross-domain stability but sacrificed colour information that could be useful for contour delineation. Future work could adopt stain normalisation or H&E dual-channel processing to achieve a better balance. Third, the weakly supervised labels generated from IHC-to-H&E conversion were not validated against expert annotations, and incorporating pathologist review would strengthen reliability. Finally, all results were reported at a fixed threshold (0.5). In clinical deployment, however, operating points should be chosen according to recall–precision trade-offs or FROC analysis; such analysis will be an important future direction.

Future research could be carried out from the following directions: first, with the accumulation of more UHS clinical data, the public dataset could be gradually replaced, so that the experiment could be carried out under the distribution of real clinical data, thereby obtaining higher reliability and applicability. Second, a real online HITL system could be explored, combining interactive labelling and a continuous learning mechanism to enable the model to continuously self-optimize in actual diagnosis. Finally, in the future, the network structure could be improved or the reinforcement learning mechanism introduced to achieve a better balance between the improvement in recall and the maintenance of precision to further improve clinical usability.

## Figures and Tables

**Table 1:** List of Human Organs and Tissues in the NuInsSeg Dataset.

Organ/Tissue	Organ/Tissue	Organ/Tissue
Human bladder	Human pancreas	Human brain
Human peritoneum	Human cardia	Human placenta
Human cerebellum	Human pylorus	Human epiglottis
Human rectum	Human jejunum	Human salivary gland
Human kidney	Human spleen	Human liver
Human testis	Human lung	Human tongue
Human melanoma	Human tonsil	Human muscle
Human umbilical cord	Human oesophagus	

**Table 2:** Number of images in the NuInsSeg dataset before and after pre-processing.

Dataset	Original Images	After Augmentation	Final Used
NuInsSeg	472	1888	Train: 1510 Test: 378
		

**Table 3:** Summary of UHS-MelHist dataset composition. Each paired sample corresponds to an H&E and Melan-A stained ROI derived from sentinel lymph node metastases.

Source ROIs (SLN)	Derived ROI Pairs	ROI Size (Pixels)
SLN_01	6	2100 × 1500
SLN_02	7	2100 × 1500
SLN_03	5	2100 × 1500
SLN_04	7	2100 × 1500
SLN_05	6	2100 × 1500
SLN_06	6	2100 × 1500
**Total**	**37**	—

**Table 4:** Number of images in the UHS-MelHist dataset before and after augmentation.

Dataset	Original Images	After Augmentation	Final Used
UHS-MelHist	37	189	Train: 144 Test: 45
		

*Note. ***“Melanoma LN”** in Table 11 refers to the same UHS-MelHist dataset introduced in this section.

**Table 5:** Hardware and software configuration of the local environment.

Component	Specification
CPU	Intel Core i7-11800H @ 2.30 GHz
RAM	16 GB
GPU	NVIDIA GeForce RTX 4090 (24 GB)
Storage	1.38 TB SSD
System	Windows 11, 64-bit
Python	3.9
PyTorch	1.12.1

**Table 6:** Confusion matrix for binary segmentation.

	Predicted Positive	Predicted Negative
**Actual Positive**	True Positive (TP)	False Negative (FN)
**Actual Negative**	False Positive (FP)	True Negative (TN)

**Table 7:** Performance comparison on NuInsSeg dataset before and after HITL.

Metric	Baseline	Post-HITL	Improvement (%)
**Recall**	0.8803	0.8983	**+2.05%**
Precision	N/A	0.6885	N/A
Dice	N/A	0.7796	N/A
IoU	N/A	0.6387	N/A

**Table 8:** Performance comparison on NuInsSeg-UHS-Combined dataset before and after HITL.

Metric	Baseline	Post-HITL	Improvement (%)
**Recall**	0.8917	0.9053 ± 0.0898	**+1.53%**
Precision	0.6869	0.7759 ± 0.1023	**+12.96%**
Dice	0.7664	0.8356 ± 0.0916	**+9.02%**
IoU	0.6433	0.7176 ± 0.1170	**+11.53%**

*Note:* Paired significance tests indicate that the HITL refinement led to a significantly higher mean performance across organs/tissues. (paired *t*-test: two-sided p<0.001; Wilcoxon signed-rank test: two-sided $p_{w}<0.001$). (95% CIs: [+0.018,+0.055], [+0.019, +0.044], and [+0.026,+0.057], respectively).

**Table 9:** Comprehensive performance and efficiency comparison on the NuInsSeg-UHS-Combined dataset. **Bold** indicates the best result, and underline indicates the second best recall.

Model	Recall	Prec	Dice	IoU	FNR (%)	Params (M)	GFLOPs	Mem (MB)	Steps/s	Epoch (s)	Imgs/s	Latency (ms)
U-Net [14]	0.8701	0.8416	0.8556	0.7476	18.6	31.04	83.70	592.1	29.68	7.70	995.7	1.00
AttentionU-Net [16]	0.8842	0.8454	0.8644	0.7611	7.3	31.39	85.35	598.7	27.53	8.30	904.0	1.11
SwinU-Net [21]	0.8380	0.8463	0.8421	0.7273	37.2	31.04	83.70	592.1	29.73	7.70	993.9	1.01
TransU-Net [17]	0.8873	0.7750	0.8273	0.7055	4.4	31.08	83.78	592.8	29.62	7.70	988.9	1.01
DeepLabV3+ [29]	0.8421	0.8268	0.8344	0.7159	35.2	3.02	36.47	57.5	51.83	4.40	1767.2	0.57
MedT [20]	**0.9562**	0.4291	0.5924	0.4208	−165.1	2.63	16.00	50.1	12.88	17.70	323.2	3.09
**ClinSegNet (Ours)**	0.8917	0.6869	0.7664	0.6433	**–**	9.79	83.90	185.3	29.70	7.70	724.3	1.38

*Note.* Metrics are averaged over the NuInsSeg-UHS-Combined test set. Prec stands for precision; FNR stands for FN Reduction rate; Params (M) denotes total learnable parameters; GFLOPs is per-image forward FLOPs (THOP-based, MACs × 2); Mem (MB) estimates parameter+optimizer memory in training; Epoch (s) assumes 1818 images (≈228 steps/epoch); Steps/s is measured training throughput; Imgs/s and Latency (ms) are inference throughput and per-image latency.

**Table 10:** Ablation study on the NuInsSeg-UHS-Combined dataset. **Bold** indicates the best result, underline indicates the second best result, and ✓ denotes the inclusion of the corresponding module. $t_{p}$ and $w_{p}$ denote the *p*-values of paired *t*-test and Wilcoxon signed-rank test, respectively.

BM	SE	FiLM	Edge	Recall (±SD)	Precision	Dice	IoU	$t_{p}$	$w_{p}$
1	–	–	–	0.8545 ± 0.012	0.8491	0.8518	0.7418	–	–
2	✓	–	–	0.8565 ± 0.015	0.7879	0.8208	0.6960	*p* = 0.0000003	*p* = 0.0000016
3	–	✓	–	0.8356 ± 0.014	0.7000	0.7618	0.6153	*p* = 0.4219	*p* = 0.0000045
4	–	–	✓	0.8615 ± 0.018	0.8484	0.8549	0.7466	*p* < 0.000001	*p* < 0.000001
5	✓	✓	–	0.8703 ± 0.011	0.8312	0.8503	0.7396	*p* < 0.000001	*p* < 0.000001
6	✓	–	✓	0.8600 ± 0.010	**0.8509**	**0.8554**	**0.7473**	*p* = 0.1158	*p* = 0.8766
7	–	✓	✓	0.8781 ± 0.013	0.7975	0.8358	0.7180	*p* = 0.0518	*p* = 0.0000002
8	✓	✓	✓	**0.8917 ± 0.009**	0.6869	0.7664	0.6433	*p* = 0.2583	*p* = 0.000587

*Note.* SD denotes the standard deviation of recall across validation subsets. Values are presented as mean ± SD. All significance tests ($t_{p}$ and $w_{p}$) are conducted against the plain U-Net baseline (BM 1, without SE, FiLM, or Edge modules). Most configurations show significance under the Wilcoxon signed-rank test ($p_{w}<0.001$), indicating consistent and robust performance improvements, even when the paired *t*-test assumptions of normality are not fully met.

**Table 11:** Per-organ recall on NuInsSeg and NuInsSeg-UHS-Combined datasets. The results show that recall generally improves after incorporating UHS samples.

Organ	NuInsSeg	Combined	Organ	NuInsSeg	Combined
Bladder	0.9510	0.9377	Liver	0.9148	0.9234
Brain	0.8032	0.8202	Lung	0.8778	0.8671
Cardia	0.8995	0.9253	Melanoma	0.7655	0.7738
Cerebellum	0.9202	0.9474	**Melanoma LN**	—	0.7460
Epiglottis	0.9049	0.9016	Muscle	0.5981	0.8175
Jejunum	0.9307	0.9475	Oesophagus	0.9165	0.9429
Kidney	0.9514	0.9437	Pancreas	0.9110	0.9184
Peritoneum	0.8385	0.8678	Placenta	0.9460	0.9453
Pylorus	0.8098	0.9013	Rectum	0.7236	0.9102
Salivary	0.8983	0.9219	Spleen	0.8796	0.9060
Testis	0.7975	0.8258	Tongue	0.8513	0.9093
Tonsil	0.9031	0.8522	Umbilical	0.7579	0.7512

*Note:* Paired significance tests indicate that the Combined setting achieved a significantly higher mean recall than NuInsSeg (paired *t*-test: two-sided p=0.024; Wilcoxon signed-rank test: two-sided p=0.004). The average improvement was +0.031 (95% CI: +0.004 to +0.057), with a medium effect size (Cohen’s $d_{z}\approx 0.50$); 16 organs improved and 7 declined.

**Table 12:** Performance comparison under different FiLM embedding dimensions. Values are presented as mean ± SD over two random seeds. The best result in each column is highlighted in **bold**.

FiLM dim	Params (M)	Recall	Dice	IoU
16	9.66	0.8616 ± 0.0057	0.7756 ± 0.0325	0.6522 ± 0.0382
32	9.79	0.8879 ± 0.0004	**0.7763** ± 0.0280	**0.6517** ± 0.0343
64	9.88	**0.8948** ± 0.0227	0.7653 ± 0.0047	0.6370 ± 0.0042
128	10.06	0.8616 ± 0.0065	0.7661 ± 0.0063	0.6388 ± 0.0062
256	10.42	0.8129 ± 0.0013	0.7440 ± 0.0176	0.6372 ± 0.0199

**Table 13:** Performance under different HistoLoss ratios (BCE:Tversky). Values are mean ± SD over two runs. The best results are highlighted in **bold**.

BCE:Tversky	Recall	Dice	IoU
0.2:0.8	0.8506 ± 0.0185	0.7658 ± 0.0016	0.6395 ± 0.0012
0.3:0.7	**0.8849** ± 0.0016	**0.7906** ± 0.0115	**0.6689** ± 0.0137
0.4:0.6	0.8403 ± 0.0300	0.7514 ± 0.0184	0.6210 ± 0.0218
0.5:0.5	0.8178 ± 0.0101	0.7150 ± 0.0337	0.5757 ± 0.0381
0.7:0.3	0.7882 ± 0.0046	0.7446 ± 0.0033	0.6116 ± 0.0042
0.8:0.2	0.7230 ± 0.0000	0.6620 ± 0.0000	0.5296 ± 0.0000

## Data Availability

Publicly available datasets were analysed in this study, which can be found at NuInsSeg (https://www.kaggle.com/datasets/ipateam/nuinsseg, accessed on 30 July 2025). The UHS-MelHist Dataset and Combined NuInsSeg-UHS Dataset contains anonymized clinical images from University Hospital Southampton (UHS) and is not publicly available due to privacy and ethical restrictions. Data access may be possible upon reasonable request to the corresponding author, subject to approval by UHS and the University of Southampton.
